# Gemcitabine radiosensitization primes irradiated malignant meningioma cells for senolytic elimination by navitoclax

**DOI:** 10.1093/noajnl/vdab148

**Published:** 2021-10-08

**Authors:** Masahiro Yamamoto, Tomomi Sanomachi, Shuhei Suzuki, Keita Togashi, Asuka Sugai, Shizuka Seino, Atsushi Sato, Masashi Okada, Chifumi Kitanaka

**Affiliations:** 1 Department of Molecular Cancer Science, Yamagata University School of Medicine, Yamagata, Japan; 2 Department of Clinical Oncology, Yamagata University School of Medicine, Yamagata, Japan; 3 Department of Ophthalmology and Visual Sciences, Yamagata University School of Medicine, Yamagata, Japan; 4 Department of Neurosurgery, Yamagata University School of Medicine, Yamagata, Japan; 5 Research Institute for Promotion of Medical Sciences, Yamagata University Faculty of Medicine, Yamagata, Japan

**Keywords:** gemcitabine, malignant meningioma, radiation, senescence, senolytics

## Abstract

**Background:**

Malignant meningioma is an aggressive tumor that requires adjuvant radiotherapy after surgery, yet there has been no standard systemic therapy established so far. We recently reported that malignant meningioma cells are highly sensitive to gemcitabine; however, it remains unknown whether or how gemcitabine interacts with ionizing radiation (IR) in malignant meningioma cells.

**Methods:**

We examined the radiosensitization effects of gemcitabine using malignant meningioma cell lines and xenografts and explored the underlying mechanisms.

**Results:**

Gemcitabine sensitized malignant meningioma cells to IR through the induction of senescence both in vitro and in vivo. Gemcitabine augmented the intracellular production of reactive oxygen species (ROS) by IR, which, together with cell growth suppression/senescence induced by this combination, was inhibited by N-acetyl-cysteine, suggesting a pivotal role for ROS in these combinatorial effects. Navitoclax, a senolytic drug that inhibits Bcl-2 proteins, further enhanced the effects of the combination of gemcitabine and IR by strongly inducing apoptotic cell death in senescent cells.

**Conclusion:**

These results not only indicate the potential of gemcitabine as a candidate radiosensitizer for malignant meningioma, but also reveal a novel role for gemcitabine radiosensitization as a means to create a therapeutic vulnerability of senescent meningioma cells to senolytics.

**Key Points**

- Gemcitabine radiosensitizes malignant meningioma cells.
- Gemcitabine cooperates with ionizing radiation to induce cellular senescence.
- Navitoclax eliminates senescent malignant meningioma cells via senolysis.

**Importance of the Study**

Treatment of malignant meningioma is among the most challenging clinical issues in neuro-oncology, with therapeutic options limited to local therapies such as surgery and radiotherapy; development of new strategies, systemic therapies, in particular, is desperately needed. Recently, we reported that malignant meningioma cells are highly sensitive to gemcitabine, but it remains unknown how gemcitabine impacts the effects of ionizing radiation on malignant meningioma cells. Here we show that gemcitabine is an efficient radiosensitizer for malignant meningioma cells. Notably, the radiosensitizing effects are mediated by cellular senescence, which is a novel mechanism of radiosensitization by gemcitabine. Furthermore, we also show navitoclax, an inhibitor of Bcl-2 family proteins, boosts the combinatorial effects of gemcitabine and radiation through its senolytic activity. Our findings suggest adding gemcitabine concomitant with radiotherapy in the treatment of malignant meningioma is a promising and rational approach, which also provides an opportunity to further improve the treatment outcome.

Meningioma is the most common primary brain tumor in adults, accounting for ~40% of intracranial neoplasms.^1^ Most meningiomas are benign, WHO grade 1,^2^ and may be successfully treated by complete resection^3^; however, a few (~20%) are high grade, WHO grade 2 or 3, with frequent recurrence and a poor prognosis.^1,4^ Malignant meningiomas are WHO grade 3 tumors that represent one of the subtypes of meningioma associated with the poorest prognosis. The standard treatment for malignant meningioma is maximal surgical resection followed by adjuvant radiation therapy.^5^ However, even after standard care, the prognosis of patients with malignant meningioma remains dismal, with 5-year recurrence-free survival rates ranging between 12 and 57%.^3^ Since there are currently no effective systemic treatments available for this subtype of meningioma,^6^ studies to improve the efficacy of radiation therapy are warranted.^7^

Gemcitabine is a pyrimidine nucleoside antimetabolite used in the treatment of several types of malignant tumors, including pancreatic cancer, non-small cell lung cancer, cholangiocarcinoma, and urothelial carcinoma. We previously identified gemcitabine as a highly effective drug against high-grade meningioma cells in vivo in xenograft models as well as in vitro^8^ and, accordingly, gemcitabine is now listed as one of the candidate therapeutic drugs for meningioma in the guidelines of the European Neuro-Oncology Association.^9^ Furthermore, we recently demonstrated that high-grade meningiomas expressed high levels of human equilibrative nucleoside transporter 1 (hENT1) and deoxycytidine kinase (dCK), which play critical roles in the cellular uptake and activation of gemcitabine, respectively, and also that high-grade meningioma cells expressing hENT1 and dCK at high levels, including malignant meningioma cells, were sensitive to gemcitabine both in vitro and in vivo.^10^ In support of these findings, a recent study reported that gemcitabine administered to a small number of patients with recurrent meningiomas on compassionate grounds achieved excellent outcomes, which underscores its potential as a therapeutic agent for aggressive meningiomas and has also led to the commencement of a clinical trial to evaluate its efficacy for recurrent high-grade meningiomas.^11^ In addition to its efficacy as a single agent, gemcitabine has been shown to act as a radiosensitizer in tumor cells both in vitro and in vivo.^12,13^ Consistent with these findings, a previous study reported that the combination of gemcitabine with ionizing radiation (IR) prolonged the survival of patients with advanced pancreatic cancer.^14^ However, it currently remains unknown whether gemcitabine enhances the effects of IR in malignant meningiomas. Therefore, we herein investigated the effects of gemcitabine combined with IR on malignant meningioma cells.

## Materials and Methods

### Cell Culture

IOMM-Lee and HKBMM, human malignant meningioma cell lines, were obtained from the American Type Culture Collection (Manassas, VA, USA) and from the Riken BioResource Center (Tsukuba, Japan), respectively. IOMM-Lee was cultured in Dulbecco’s modified Eagle’s medium (DMEM) supplemented with 10% fetal bovine serum (FBS). HKBMM was cultured in Ham’s F12 medium supplemented with 10% FBS.

### Mouse Study

After assessing cell viability using the dye exclusion method, 1 × 10^6^ viable IOMM-Lee cells or 2 × 10^6^ viable HKBMM cells were suspended in 100 µL PBS and implanted in the flank regions of 5- to 7-week-old male BALB/cAJcl-*nu/nu* mice (CLEA Japan, Tokyo, Japan) anesthetized by a subcutaneous injection of medetomidine, midazolam, and butorphanol (0.3, 4, and 5 mg per kg body weight, respectively). Tumor volumes were assessed by measuring tumor diameters with a digital caliper and calculated using the following formula: (length) × (width) × (depth) × π/6. After the average volume of tumors reached greater than 100 mm^3^ for IOMM-Lee or 50 mm^3^ for HKBMM, mice were randomized according to tumor volumes and then treated with gemcitabine (dissolved in PBS, 10 mg/kg body weight, intraperitoneal injection), IR (1 Gy), navitoclax (dissolved in 20% DMSO and 80% corn oil, 100 mg/kg body weight, oral gavage), their vehicles (Control), or their combination.

The intracranial implantation of IOMM-Lee cells was performed as previously described.^10^ Briefly, after 5-week-old male BALB/cAjcl-*nu/nu* mice (CLEA, Japan) had been anesthetized and fixed in a stereotactic frame (Narishige, Tokyo, Japan), a burr hole was made in the parietal bone 2 mm posterior and 2 mm lateral to the bregma. IOMM-Lee cells suspended in PBS (2 × 10^5^ in 2 µL) were intracranially implanted through the burr hole 4.0 mm below the skull surface with a 25-µL Hamilton syringe (Hamilton, Reno, NV, USA) that was mounted onto the stereotactic frame.

### Ionizing Radiation

Ionizing irradiation was conducted at a dose rate of 1 Gy/min using TITAN-225S (Shimazu Systems, Shiga, Japan) in the animal facility of Yamagata University. Regarding the radiation treatment, mice were anesthetized and protected by a 6-mm-thick lead shield, except for the abdominal region bearing subcutaneous tumors for the subcutaneous tumor model or the head for the intracranial tumor model.

### Study Approval

The Animal Research Committee of Yamagata University approved all animal experiments.

### Statistics

Data were analyzed using a 1-way analysis of variance (ANOVA) followed by Tukey’s or Sidak’s post hoc test, the Kruskal-Wallis test followed by Dunn’s multiple comparison test, the Brown-Forsythe and Welch ANOVA tests, or a 2-way ANOVA with the Bonferroni multiple comparisons test. The survival of mice in an intracranial model was compared using the Log-rank test. A *P*-value of <.05 was considered to be significant. All analyses were performed using GraphPad Prism version 9 for Mac (GraphPad Software, San Diego, CA, USA).

The detail of materials and other methods are described in the Supplementary Material.

## Results

### Gemcitabine Enhances Growth-Suppressive Effects of Ionizing Radiation in Malignant Meningioma Cells In Vitro

**Figure 1.**
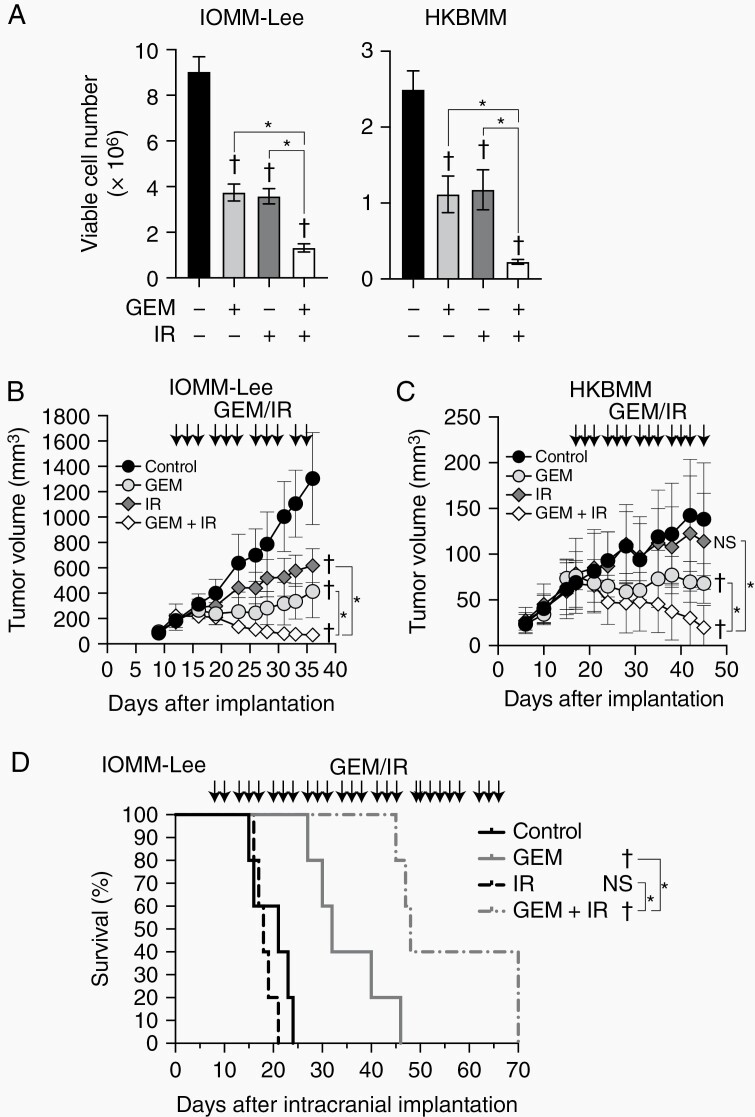
Combined effects of gemcitabine with ionizing radiation on malignant meningioma cells in vitro and in vivo. (A) IOMM-Lee and HKBMM cells plated on 6-well plates in 6 replicates (2 × 10^5^ cells per well for IOMM-Lee and 4 × 10^5^ cells per well for HKBMM) were incubated without or with gemcitabine (3 nM for IOMM-Lee and 2 nM for HKBMM) for 6 days and not irradiated or irradiated 3 times by X-ray (1 Gy for IOMM-Lee and 2 Gy for HKBMM) on days 1, 3, and 5, and subjected to a cell viability assay. (B and C) IOMM-Lee and HKBMM cells were subcutaneously implanted in the flank regions bilaterally (1 × 10^6^ for IOMM-Lee and 2 × 10^6^ for HKBMM). After tumor establishment was confirmed, mice were treated with gemcitabine (10 mg/kg, intraperitoneal injection) (GEM), ionizing radiation (1 Gy) (IR), both (GEM+IR), or vehicle (Control) 3 times a week (arrows). The size of tumors was measured (n = 8 for each group). (D) Eight days after the intracranial implantation of IOMM-Lee cells (2 × 10^5^ cells per mouse), mice were treated with gemcitabine (10 mg/kg) (GEM), ionizing radiation (1 Gy) (IR), both (GEM + IR), or vehicle (Control) 3 times a week (arrows). The survival of mice was shown as the Kaplan-Meier plot (n = 5, each group). GEM, gemcitabine. IR, ionizing radiation. Values are shown as mean ± SD. In (A), (B), and (C), *P*-values were calculated by a 1-way ANOVA with Tukey’s post hoc test. In (D), *P*-values were calculated by the Log-rank test. *P < .05. ^†^*P* < .05 and NS, *P* ≥ .05 vs the Control (GEM− and IR−).

To investigate whether gemcitabine affects the tumor-suppressive effects of IR in malignant meningioma cells, we performed screening experiments using the WST-8 assay. The combination of radiation and gemcitabine decreased cell viability more than either treatment alone at a certain range of gemcitabine concentrations and radiation doses in IOMM-Lee and HKBMM malignant meningioma cells (Supplementary Figure S1). Accordingly, in subsequent experiments, we selected concentrations (3 and 2 nM) of gemcitabine and doses (1 and 2 Gy) of IR to treat IOMM-Lee and HKBMM, respectively. The combination of gemcitabine and radiation suppressed cell growth more than either treatment alone in both IOMM-Lee and HKBMM cells (Figure 1A). Furthermore, the combination suppressed colony formation activity more than either treatment alone (Supplementary Figure S2A and B). These results suggest that gemcitabine enhances the growth-suppressive effects of IR on malignant meningioma cells in vitro.

### Gemcitabine Enhances Tumor-Suppressive Effects of Ionizing Radiation in Malignant Meningioma Cells In Vivo

To evaluate the potential clinical significance of the combination of gemcitabine and IR in malignant meningioma, we examined their effects in subcutaneous and intracranial malignant meningioma models. In the IOMM-Lee subcutaneous tumor model, gemcitabine and IR each suppressed tumor growth, and their combination inhibited tumor growth more than either treatment alone (Figure 1B). In the HKBMM subcutaneous tumor model, IR alone failed to suppress tumor growth, while gemcitabine exerted inhibitory effects as previously reported.^8^ However, again, the combination of gemcitabine and IR suppressed tumor growth more than either treatment alone, similar to the IOMM-Lee model (Figure 1C). Furthermore, in the intracranial meningioma model, gemcitabine, but not IR, prolonged the survival of mice when used alone, while their combination prolonged survival significantly more than either treatment alone (*P* = .0018 vs the IR-treated group and *P* = .0064 vs the gemcitabine-treated group, Figure 1D). These results indicate that the combination of gemcitabine and IR is effective in preclinical malignant meningioma models.

### Combination of Gemcitabine and Ionizing Radiation Induces Cellular Senescence in Malignant Meningioma Cells In Vitro

**Figure 2.**
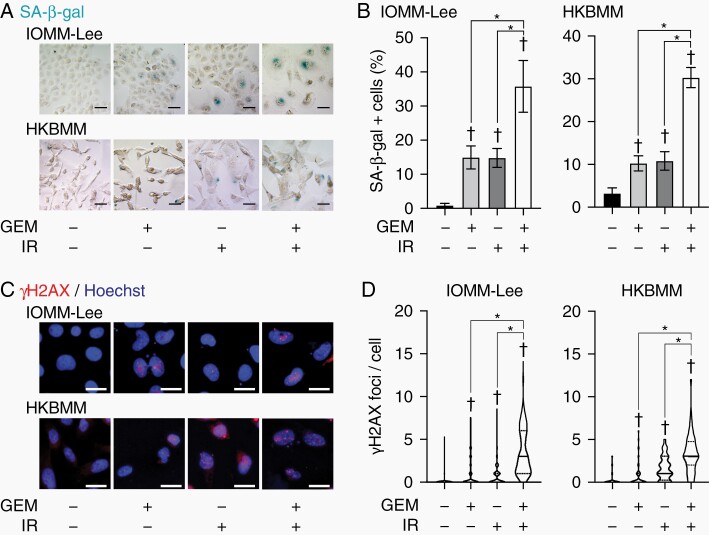
Induction of senescence markers by gemcitabine and ionizing radiation. IOMM-Lee and HKBMM cells were incubated without or with gemcitabine (3 nM for IOMM-Lee and 2 nM for HKBMM) for 4 days and not irradiated or irradiated twice by X-ray (1 Gy for IOMM-Lee and 2 Gy for HKBMM) on days 1 and 3, and then subjected to SA-β-gal staining (A) or immunocytochemistry for γH2AX (C) on day 4. The percentage of SA-β-gal-positive cells was quantified (n = 4, each group) (B). The number of γH2AX foci was counted (>60 cells were counted for each group) and shown as violin plots (D). GEM, gemcitabine. IR, ionizing radiation. Scale bars, 50 µm in (A) and 20 µm in (C). In (B), values are shown as mean ± SD. In (D), lines represent medians and dotted lines represent quartiles. *P*-values were calculated by a 1-way ANOVA with Tukey’s post hoc test (B) or by the Kruskal-Wallis test with Dunn’s multiple comparisons test (D). *P < .05. ^†^*P* < .05 vs the Control (GEM− and IR−).

We then investigated the mechanisms underlying the radiosensitizing effects of gemcitabine in meningioma cells. Since apoptotic cell death is one of the mechanisms responsible for the radiosensitizing effects of gemcitabine,^15,16^ we examined the effects of their combination on apoptotic cell death. However, their combination did not induce cell death or apoptosis in malignant meningioma cells (Supplementary Figure S3). The results obtained showed that their combination induced characteristic morphological changes in cells, namely, an enlarged, flattened, and multinucleated morphology (Supplementary Figure S4). Since these morphological changes are the features of cells undergoing cellular senescence,^17,18^ we examined senescence-associated β-galactosidase (SA-β-gal) activity, which is a gold standard marker for senescent cells. Gemcitabine and IR increased the number of SA-β-gal-positive cells, and their combination increased this number more than either treatment alone (Figure 2A and B). Furthermore, since increased DNA damage and the enhanced production of inflammatory cytokines (termed senescence-associated secretory phenotype [SASP]) are also hallmarks of senescent cells,^19^ we examined γH2AX foci, a marker of DNA damage, and the mRNA expression of cytokines in cells. The number of γH2AX foci and the gene expression of cytokines were increased in cells treated with the combination (Figure 2C and D; Supplementary Figure S5). These results suggest that the combination of gemcitabine and radiation induces cellular senescence in malignant meningioma cells in vitro.

### Reactive Oxygen Species (ROS) Are Involved in Senescence Induced by Gemcitabine and Ionizing Radiation in Malignant Meningioma

**Figure 3.**
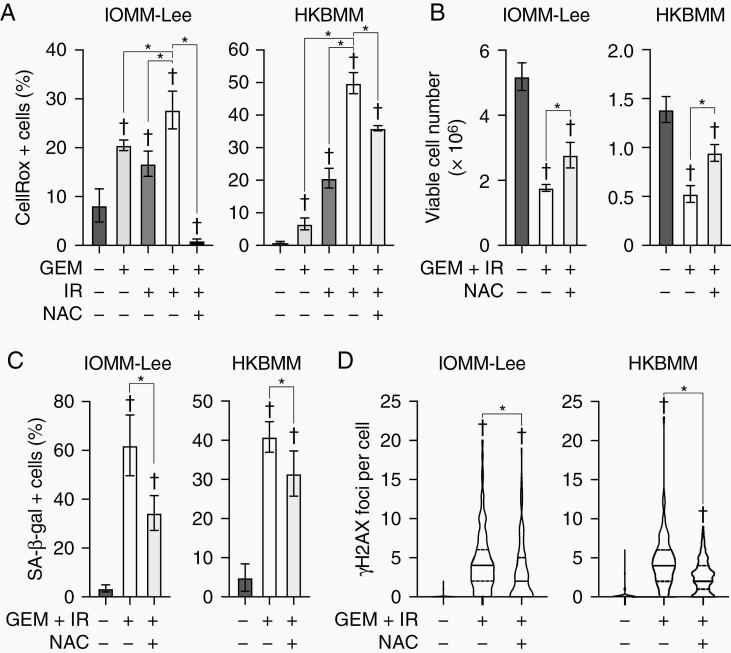
Involvement of reactive oxygen species in senescence induced by gemcitabine and ionizing radiation. IOMM-Lee and HKBMM cells plated on 6-well plates (2 × 10^5^ for IOMM-Lee and 4 × 10^5^ for HKBMM per each well) were incubated without or with gemcitabine (GEM, 3 nM for IOMM-Lee, and 2 nM for HKBMM) in the absence or presence of N-acetyl-cysteine (NAC, 5 mM) for 4 days and not irradiated or irradiated twice by X-ray (IR, 1 Gy for IOMM-Lee and 2 Gy for HKBMM) on days 1 and 3, and then subjected to a CellRox assay (A), cell viability assay (B), SA-β-gal staining (C), or γH2AX immunocytochemistry (D) on day 4. In (A), (B), and (C), experiments were performed in 4, 6, and 4 replicates, respectively, and values are shown as mean ± SD. In (D), the number of γH2AX foci was counted in more than 60 cells per group and shown as violin plots (line, median; dotted lines, quartile). *P*-values were calculated by a 1-way ANOVA with Tukey’s post hoc tests (A, B, and C) or by the Kruskal-Wallis test with Dunn’s multiple comparisons test (D). *P < .05. ^†^*P* < .05 vs the Control (no treatment).

We next attempted to elucidate the mechanisms by which the combination of gemcitabine and IR induces senescence in malignant meningioma cells. Since the increased production of ROS is one of the causes of cellular senescence induced by cancer therapy,^20,21^ their involvement was investigated. In IOMM-Lee and HKBMM cells, gemcitabine and IR enhanced the production of ROS, with the combination further promoting their production, and these increases were mitigated by a treatment with N-acetyl-cysteine (NAC), a ROS scavenger (Figure 3A). The NAC treatment partially canceled the suppression of cell growth by the combination of gemcitabine and IR (Figure 3B). In addition, the increases observed in the number of SA-β-gal-positive cells and γH2AX foci by the combination were attenuated by the NAC treatment (Figure 3C and D). These results indicate that cellular senescence induced by gemcitabine and IR is partially mediated by the mechanism enhancing ROS production.

### Combination of Gemcitabine and Ionizing Radiation Enhances Cellular Senescence in Malignant Meningioma Cells In Vivo

**Figure 4.**
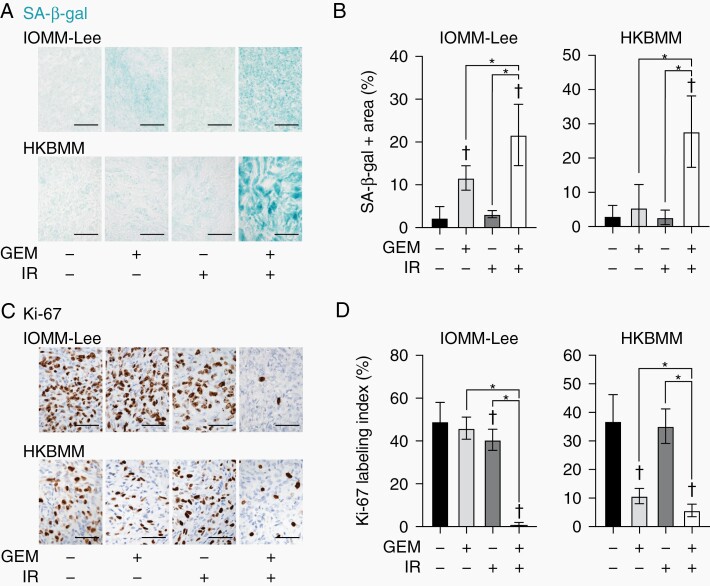
Induction of senescence markers by gemcitabine and radiation in malignant meningioma subcutaneous xenografts. Representative images of SA-β-gal staining (A) and Ki-67 immunohistochemistry (C) in excised IOMM-Lee and HKBMM xenografts. Scale bars, 50 µm. The percentage of the SA-β-gal-positive area (B) and Ki-67 labeling index (D) were quantified. GEM, gemcitabine. IR, ionizing radiation. In (B), IOMM-Lee: Control, n = 5; GEM, n = 5; IR, n = 5; GEM + IR, n = 6, HKBMM: Control, n = 6; GEM, n = 5; IR, n = 8; GEM + IR, n = 5. In (D), IOMM-Lee: n = 8 for each group, HKBMM: Control, n = 8; GEM, n = 4; IR, n = 8; GEM + IR, n = 4. Values are shown as mean ± SD. *P*-values were calculated by a 1-way ANOVA with Tukey’s post hoc test. *P < .05. ^†^*P* < .05 vs the Control (GEM− and IR−).

To clarify whether the mechanisms underlying the combined effects of gemcitabine and IR in vivo are also mediated by cellular senescence, SA-β-gal activity was examined in the malignant meningioma subcutaneous models. In IOMM-Lee and HKBMM models, the combination of gemcitabine and IR enhanced SA-β-gal activity in malignant meningioma cells more than either treatment alone (Figure 4A and B). Since a low Ki-67 proliferation index has been identified as an in vivo marker of cellular senescence,^18^ we examined Ki-67 in subcutaneous tumors. The Ki-67 labeling index was lower in malignant meningioma cells treated with the combination of gemcitabine and IR than in those with either treatment alone (Figure 4C and D). These results suggest that the combination of gemcitabine and IR also enhances cellular senescence in preclinical malignant meningioma models.

### Navitoclax Enhances Combinational Effects of Gemcitabine and Ionizing Radiation in Malignant Meningioma Cells In Vitro

**Figure 5.**
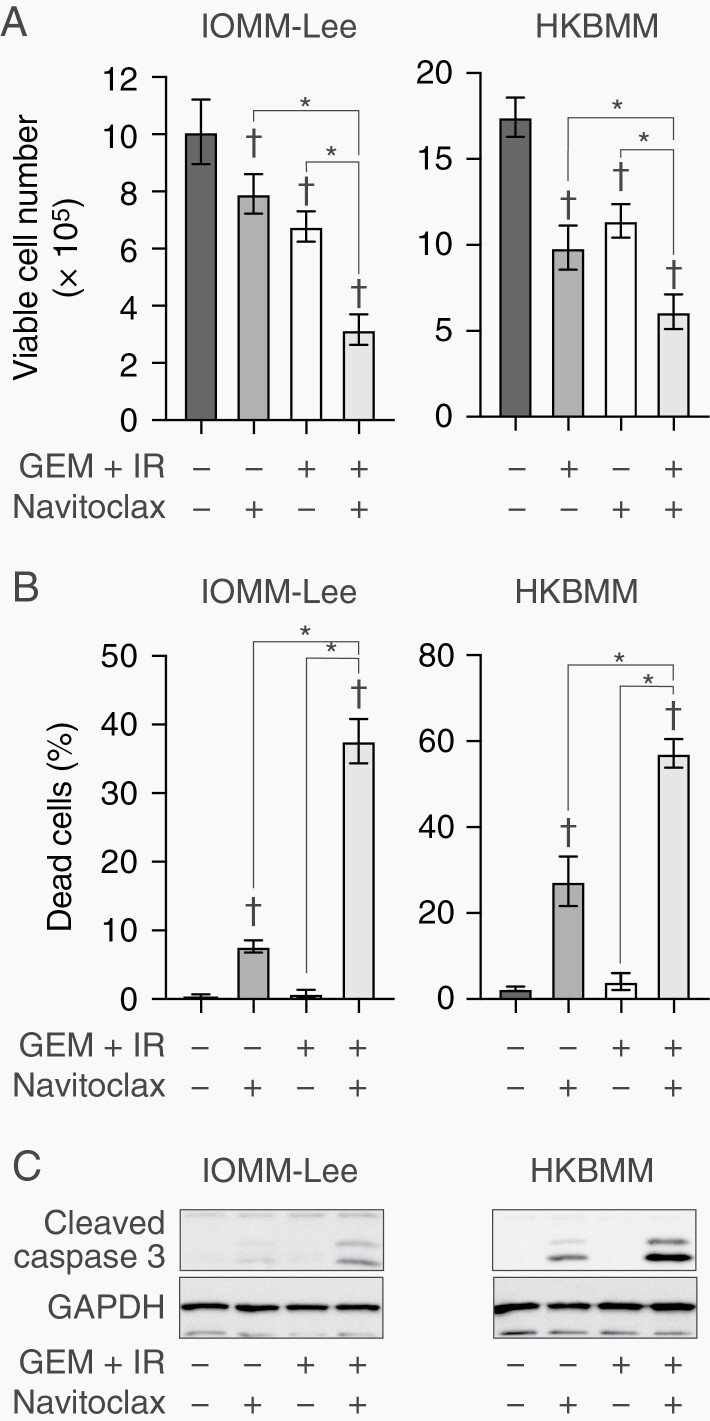
Effects of navitoclax combined with gemcitabine and ionizing radiation on malignant meningioma cells. IOMM-Lee and HKBMM cells plated on 6-well plates in 6 replicates (2 × 10^5^ cells per well for IOMM-Lee and 4 × 10^5^ cells per well for HKBMM) were incubated for 2 days without or with gemcitabine (3 nM for IOMM-Lee and 2 nM for HKBMM) in combination with ionizing radiation (1 Gy for IOMM-Lee and 2 Gy for HKBMM) on day 1 in the absence or presence of navitoclax (1 µM) and were then subjected to a cell viability assay to assess the viable cell number (A) and percentage of dead cells (B) or to a Western blot analysis to examine the expression of indicated proteins (C) on day 2. Values are shown as mean ± SD. *P*-values were calculated by a 1-way ANOVA with Tukey’s post hoc test. *P < .05. ^†^*P* < .05 vs the Control (GEM + IR− and Navitoclax−).

The combination of gemcitabine and radiation induced cellular senescence and suppressed tumor growth in malignant meningioma cells. Senescence induced by therapy, termed therapy-induced senescence, is considered to be a reversible process, and thus, senescent cells retain the potential to regrow if they are not completely eliminated.^22^ After the treatment with gemcitabine and IR had been stopped, malignant meningioma cells started to grow again (Supplementary Figure S6B). Regrowth was accompanied by decreases in the senescent markers, SA-β-gal and γH2AX (Supplementary Figure S6C and D). Senolytic drugs, which selectively induce cell death in senescent cells, are candidate drugs for the elimination of senescent cancer cells induced by therapy. Several drugs reportedly have the potential to be senolytics, including dasatinib combined with quercetin,^23^ navitoclax (an inhibitor of Bcl-2 family proteins),^24^ inhibitors of the bromodomain and extraterminal domain (BET),^25^ and heat shock protein (HSP) inhibitors.^26^ Therefore, to establish strategies that enhance the therapeutic effects of the combination of gemcitabine and IR in malignant meningioma, we screened the combined effects of senolytic drugs with gemcitabine and IR in malignant meningioma cells. Although all senolytics exhibited combined effects, those of navitoclax were the strongest (Supplementary Figure S7). Therefore, we focused on navitoclax in subsequent experiments. The combination of navitoclax with gemcitabine and IR suppressed the growth of IOMM-Lee and HKBMM cells (Figure 5A; Supplementary Figure S8). Navitoclax strongly induced cell death mediated by apoptosis in gemcitabine and radiation-treated meningioma cells (Figure 5B and C). Furthermore, navitoclax delayed the recovery of malignant meningioma cells from growth suppression induced by gemcitabine and IR (Supplementary Figure S9). Therefore, navitoclax has the potential to enhance tumor-suppressive effects in combination with gemcitabine and IR by inducing apoptotic cell death.

### Inhibition of Bcl-xL Is Involved in Senolytic Effects of Navitoclax in Senescent Malignant Meningioma Cells Treated With Gemcitabine and Ionizing Radiation

Since the main target molecules of navitoclax are Bcl-2 and Bcl-xL, we investigated which is the primary target of navitoclax in senescent malignant meningioma cells. The treatment with gemcitabine and radiation did not consistently alter the expression of Bcl-2 and Bcl-xL in malignant meningioma cells (Supplementary Figure S10A). It is important to note here that the effects of venetoclax, a Bcl-2-specific inhibitor, in combination with gemcitabine and IR were markedly weaker than those of navitoclax (Supplementary Figure S7), suggesting that the senolytic effects of navitoclax are mediated by the inhibition of Bcl-xL rather than Bcl-2. In support of this result, the suppression of Bcl-xL expression by siRNA and the inhibition of Bcl-xL by A-1331852, a Bcl-xL-specific inhibitor, sensitized malignant meningioma cells to the combination of gemcitabine and radiation (Supplementary Figures S10B, C and S11). These results suggest that Bcl-xL is the main mediator of the senolytic effects of navitoclax on senescent meningioma cells induced by the combination of gemcitabine and IR.

### Navitoclax Enhances Combinational Effects of Gemcitabine and Ionizing Radiation In Vivo

**Figure 6.**
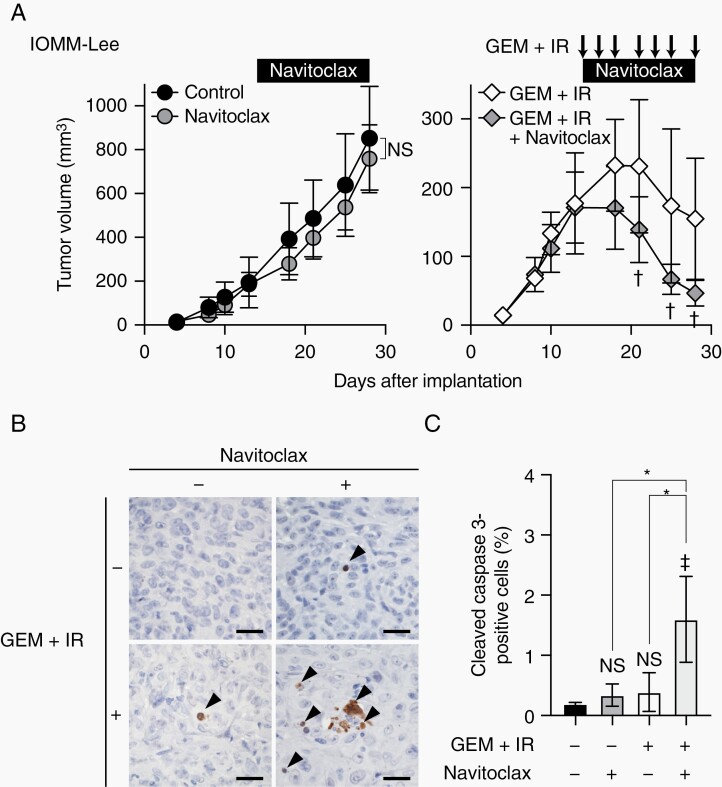
Effects of navitoclax on malignant meningioma xenografts in combination with gemcitabine and ionizing radiation. (A) IOMM-Lee cells were subcutaneously implanted in the flank regions bilaterally (1 × 10^6^). After tumor establishment was confirmed, mice were treated with navitoclax (100 mg/kg, oral gavage, every day), gemcitabine (10 mg/kg, intraperitoneal injection) in combination with ionizing radiation (1 Gy) (3 times a week, arrows) (GEM + IR), both (GEM + IR + Navitoclax), or vehicle (Control). The size of tumors was measured. Values are shown as mean ± SD (n = 8 for each group). (B) Immunohistochemistry for cleaved caspase 3 in tumors excised 1 day after the last treatment. Arrowheads indicate cleaved caspase 3-positive cells. Scale bars, 50 µm. (C) Quantification of the percentages of cleaved caspase 3-positive cells. In (A), *P*-values were calculated by a 2-way ANOVA with the Bonferroni multiple comparisons test. NS, *P* ≥ .05 and ^†^*P* < .05 vs GEM + IR. In (C), *P*-values were calculated by a 1-way ANOVA with Tukey’s post hoc test. *P < .05. ^‡^*P* < .05 and NS, *P* ≥ .05 vs the Control (no treatment).

We then investigated the clinical relevance of the navitoclax treatment combined with gemcitabine and IR in malignant meningioma. In the subcutaneous malignant meningioma model, navitoclax enhanced the suppressive effects of the combination of gemcitabine and IR on tumor growth, whereas navitoclax alone did not inhibit tumor growth (Figure 6A). A histological examination showed that navitoclax increased cleaved caspase 3-positive apoptotic cells in gemcitabine and radiation-treated malignant meningioma cells (Figure 6B and C). These results suggest that navitoclax enhances the growth-suppressive effects of gemcitabine and IR on malignant meningioma cells in vivo as well as in vitro by increasing apoptotic cell death.

## Discussion

Despite surgical resection and radiation therapy, the prognosis of malignant meningioma remains poor. Several clinical trials on systemic therapy have been performed; however, there is currently no approved standard systemic therapy for patients with meningioma.^6^ Radiation is the only non-surgical therapeutic option for malignant meningioma, and thus, strategies to enhance the effects of radiation are required. In the present study, we demonstrated that gemcitabine radiosensitized malignant meningioma cells through the mechanism of cellular senescence and that, based on this mechanism, navitoclax enhanced the therapeutic effects of the combination of gemcitabine and radiation via senolytic activity.

The mechanisms underlying the radiosensitization of cancer cells by gemcitabine have not yet been fully elucidated, except for the contribution of cell cycle redistribution, deoxynucleotide depletion, and increased DNA damage.^27^ Although increased apoptosis has been reported as a mechanism,^15,16^ the extent to which it contributes to radiosensitization by gemcitabine remains unknown. We herein demonstrated that irradiated malignant meningioma cells underwent overt senescence in the presence of gemcitabine, and thus, to the best of our knowledge, this is the first study to demonstrate the involvement of cellular senescence in the mechanisms responsible for radiosensitization by gemcitabine. Since senescent cancer cells may be targeted by senolytic drugs, as was also shown in the present study, our results provide important insights into the therapeutic potential of senolytic drugs not only in malignant meningioma but also in various types of tumors treated with gemcitabine and radiation, in the context of therapy-induced senescence discussed below.

Cellular senescence is a unique form of durable cell growth arrest that is accompanied by characteristic changes, such as a flat and enlarged morphology, increased lysosomal β-galactosidase activity, and the secretion of pro-inflammatory cytokines.^19^ Cellular senescence is induced by various cellular stresses, including telomere shortening, oncogene activation, oxidative stress, and DNA damage.^28^ In the present study, we showed that elevated ROS levels, at least in part, play a role in increased DNA damage and cellular senescence caused by the combination of gemcitabine and IR in malignant meningioma cells. Consistent with the present results, cellular senescence caused by gemcitabine was mediated by oxidative stress in pancreatic cancer cells.^20^ Furthermore, IR indirectly induced DNA damage mediated by increased ROS levels along with direct DNA damage, resulting in cellular senescence.^21^ Previous studies also demonstrated that gemcitabine prevented DNA repair by inhibiting DNA polymerase and Rad51-dependent DNA repair^27,29^; therefore, impaired DNA repair by gemcitabine may also contribute to increased DNA damage in senescent meningioma cells. Collectively, these results suggest that cellular senescence induced by the combination of gemcitabine and IR in malignant meningioma cells was mediated by increased ROS production and the subsequent accumulation of DNA damage, which may be further promoted by impaired DNA damage repair due to gemcitabine as well as by the direct effects of IR.

Besides these mechanistic insights, our demonstration of the gemcitabine-mediated radiosensitization of malignant meningioma cells also has significant therapeutic implications because, even though radiosensitization strategies for cancer have been investigated,^30^ only a few drugs have been reported for meningioma. LB-100, an inhibitor of protein phosphatase 2A, has been shown to sensitize malignant meningioma cells to radiation.^31^ Valproic acid promotes radiosensitization in meningioma stem-like cells.^32^ Mebendazole, an antiparasitic widely used to treat helminth infections, was found to increase the survival of rodents treated with radiation in an intracranial model of malignant meningioma.^33^ Malignant meningioma is treated with various forms of radiation therapy, including fractioned external beam radiation therapy (EBRT), stereotactic radiosurgery (SRS), and brachytherapy.^6^ Previous studies examined the in vivo effects of LB-100 or mebendazole on fractioned EBRT or SRS, respectively.^31,33^ In the present study, we mainly used radiation methods mimicking fractioned EBRT. Although gemcitabine enhanced the tumor-suppressive effects of single high-dose radiation (6 Gy for IOMM-Lee and 8 Gy for HKBMM) in vitro (Supplementary Figure S1), further studies are warranted to examine the enhancement by gemcitabine of single high-dose radiation mimicking SRS in vivo. Notably, studies on these potential radiosensitizers have also been conducted on humans. LB-100 was tolerated well by patients with advanced solid tumors in a phase 1 clinical trial (NCT01837667); however, its efficacy in patients with cancer is still under investigation in phase 2 clinical trials (NCT03027388, NCT03886662). Therefore, LB-100 is not yet clinically available. Since valproic acid is used as an anti-epileptic drug for patients with brain tumors, its efficacy has mainly been examined in patients with glioblastoma. However, its clinical benefit as a radiosensitizer remains controversial.^34–36^ Although the safety profile of mebendazole has mainly been confirmed in patients with helminth infections and also in those with glioma in phase 1 clinical trials,^37^ its clinical effects as a radiosensitizer remain unknown.^38^ In contrast to these drugs, gemcitabine is approved for the treatment of several types of cancers, and its safety for cancer patients is widely known. Furthermore, gemcitabine is one of the drugs that have been shown to enhance the effects of radiation therapy, and its clinical benefit in chemoradiation therapy has been documented in patients with pancreatic cancer.^14^ Therefore, the combination of gemcitabine with IR may so far be the most promising among the potential therapeutic strategies for the treatment of malignant meningioma, and clinical trials to test its efficacy in malignant meningioma are warranted.

Importantly, we not only demonstrated that gemcitabine effectively radiosensitized malignant meningioma cells, but we also showed that this combination strongly induced cellular senescence, thereby providing a new opportunity for another layer of combination therapy. Cellular senescence is one of the tumor cell responses to chemotherapy and radiation, and this type of senescence is termed therapy-induced senescence. Based on the traditional understanding of senescence as an irreversible process, therapy-induced senescence was considered to be a favorable response to therapy. However, the accumulation of recent evidence demonstrated that therapy-induced senescence is not a permanent process, and senescent cells have the potential to regrow and contribute to tumor recurrence.^39,40^ Senolytics are drugs that selectively kill senescent cells in cancer treatment and the aging process.^41,42^ In the present study, we demonstrated that navitoclax, known as a senolytic drug, enhanced apoptotic cell death in senescent meningioma cells induced by gemcitabine and IR in vitro and in an in vivo subcutaneous model. Although we examined the effects of navitoclax in an intracranial model, navitoclax failed to extend the survival of mice treated with gemcitabine and IR. In our intracranial model of meningioma, meningioma cells were implanted at the skull base to recapitulate meningioma tissues; however, the implanted meningioma cells aggressively invaded brain tissues and appeared to be fed mainly by the blood vessels of brain tissues rather than extra-axial blood vessels devoid of the blood-brain barrier (data not shown). Since navitoclax does not cross the blood-brain barrier,^43^ its intra-tumoral concentration in the intracranial model was unable to reach a sufficient level to exert therapeutic effects. Due to this limitation, it currently remains unclear whether navitoclax enhances the effects of gemcitabine and IR in an intracranial model. We need to improve the model to recapitulate human meningioma tissues more closely in future studies. Still, navitoclax is expected to exert combined effects with gemcitabine and IR in humans as demonstrated in the subcutaneous meningioma model, since meningioma tissues in humans are outside the blood-brain barrier.

Navitoclax is a synthetic BH3 mimetic that binds to the BH3 domain of Bcl-2 family proteins. It relocates pro-apoptotic BH3-only proteins, such as Bim, from anti-apoptotic Bcl-2 proteins, resulting in the induction of apoptosis.^44^ The present results suggest that senolytic activity by navitoclax is, at least in part, mediated by the inhibition of Bcl-xL in malignant meningioma cells under the combined treatment with gemcitabine and IR. In accordance with the present results, navitoclax has been shown to exhibit senolytic activity in therapy-induced senescent cancer cells mainly through the inhibition of Bcl-xL.^45–47^ Collectively, the present results suggest that the combination of gemcitabine and radiation induces senescence and thereby increases the dependence of malignant meningioma cells on Bcl-xL for their survival, which is consistent with previous findings,^46,47^ while navitoclax enhanced the tumor-suppressive effects of gemcitabine and radiation by targeting Bcl-xL to induce apoptotic cell death in senescent malignant meningioma cells.

It is important to note that thrombocytopenia is a dose-limiting toxicity of navitoclax caused by the induction of apoptosis in platelets, the survival of which is largely dependent on Bcl-xL.^48,49^ In the present study, the combination of navitoclax, gemcitabine, and IR induced thrombocytopenia, but did not cause adverse effects such as hemorrhagic diathesis, weight loss, and death (Supplementary Figure S12). Venetoclax is a Bcl-2-specific inhibitor that was developed to avoid this toxicity and is used to treat hematological malignancies without thrombocytopenia.^50^ However, based on our results showing that venetoclax failed to exhibit apparent senolytic activity in senescent malignant meningioma cells, the targeting of Bcl-xL, not Bcl-2, is considered to be important for the induction of apoptosis in senescent meningioma cells. Recent studies demonstrated that novel strategies, such as proteolysis-targeted chimera (PROTAC) technology and galacto-conjugation, successfully increased the specificity of senolytic activity against tumor cells and reduced platelet toxicity.^51–53^ By utilizing these strategies, the therapeutic effects of gemcitabine and IR in meningioma may be enhanced without significant side effects.

In conclusion, gemcitabine suppressed the growth of malignant meningioma cells in combination with IR by inducing cellular senescence, and thus, has emerged as a novel potential radiosensitizer for patients with malignant meningioma. Furthermore, their combined effects may be further enhanced by inhibiting Bcl-xL with senolytics, such as navitoclax.

## Supplementary Material

vdab148_suppl_Supplementary_Figure_S1

vdab148_suppl_Supplementary_Figure_S10

vdab148_suppl_Supplementary_Figure_S11

vdab148_suppl_Supplementary_Figure_S12

vdab148_suppl_Supplementary_Figure_S2

vdab148_suppl_Supplementary_Figure_S3

vdab148_suppl_Supplementary_Figure_S4

vdab148_suppl_Supplementary_Figure_S5

vdab148_suppl_Supplementary_Figure_S6

vdab148_suppl_Supplementary_Figure_S7

vdab148_suppl_Supplementary_Figure_S8

vdab148_suppl_Supplementary_Figure_S9

vdab148_suppl_Supplementary_Material

## Notes

**Authorship Statement**. Conceptualization: M.Y. and C.K. Project administration: C.K. Supervision: C.K. Investigation and validation: M.Y. Funding acquisition: T.S. Resources: A.Sugai and S.Seino. Writing—original draft: M.Y. and C.K. Writing—review and editing: M.Y., T.S., S.Suzuki, K.T., A.Sugai, S.Seino, A.Sato, M.O., and C.K.
