# Supplementary figures and images for: Gemcitabine radiosensitization primes irradiated malignant meningioma cells for senolytic elimination by navitoclax
Source: Neurooncol Adv. 2021 Oct 8;3(1):vdab148. doi: 10.1093/noajnl/vdab148 (PMC8577526; doi:10.1093/noajnl/vdab148)

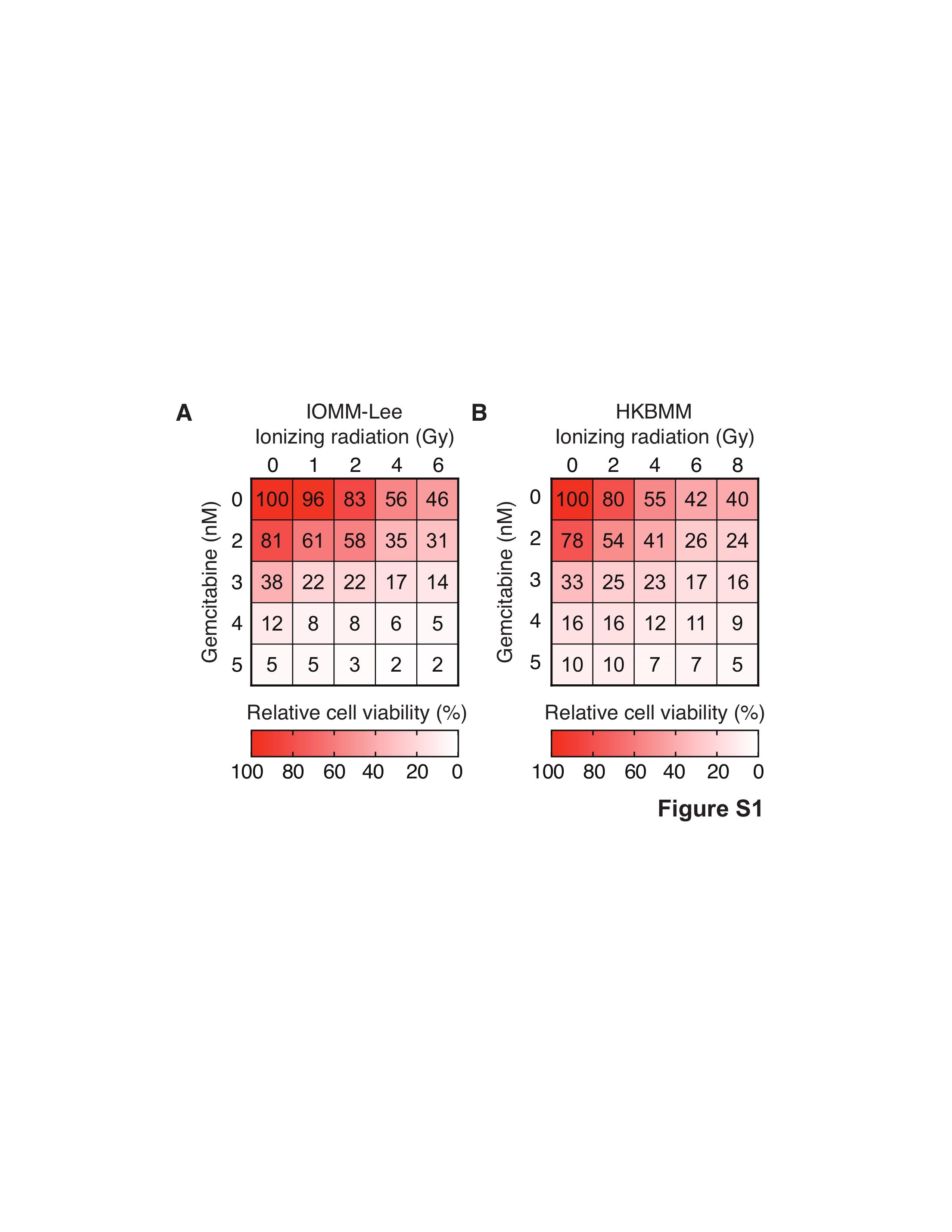

Supplement: vdab148_suppl_Supplementary_Figure_S1 [file vdab148_suppl_supplementary_figure_s1.jpeg]

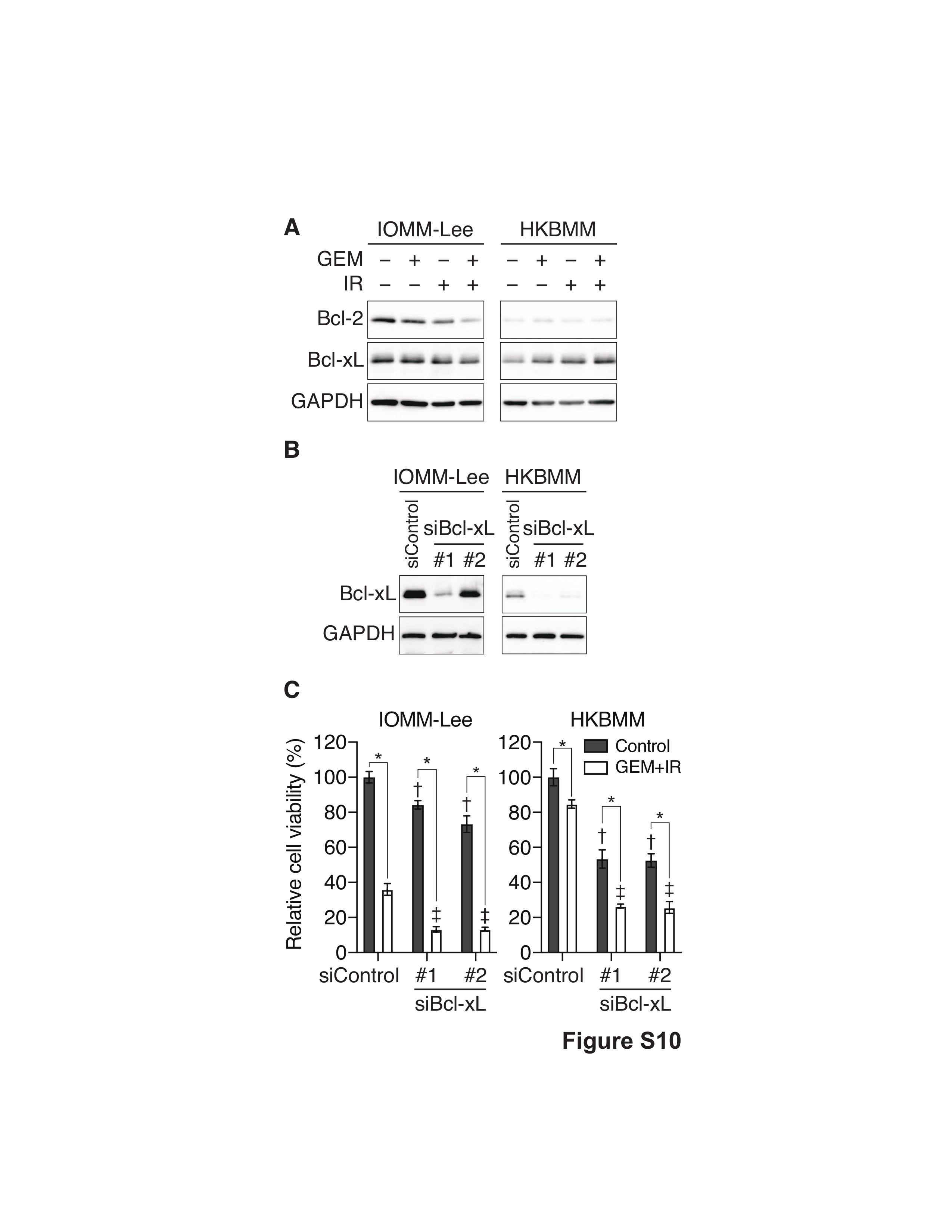

Supplement: vdab148_suppl_Supplementary_Figure_S10 [file vdab148_suppl_supplementary_figure_s10.jpeg]

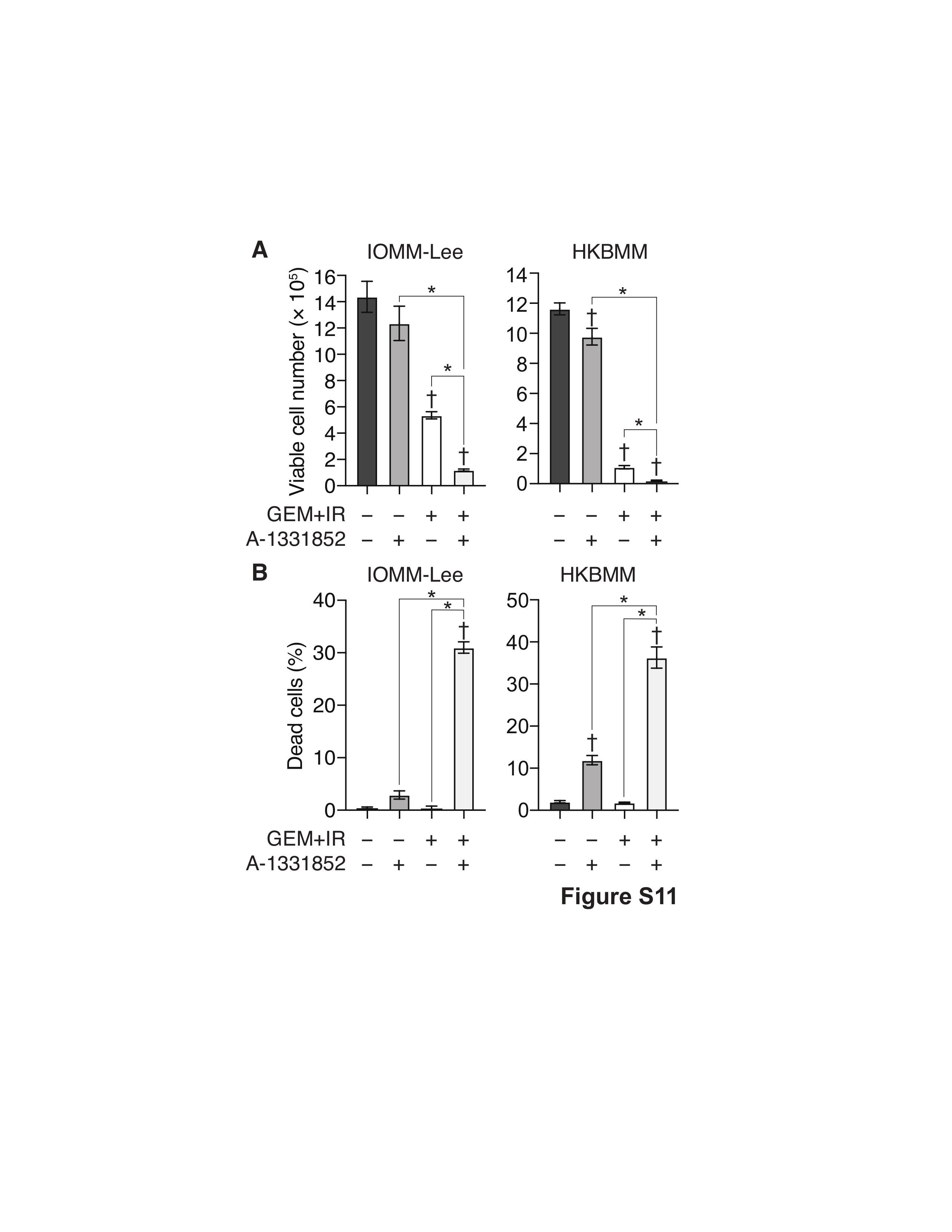

Supplement: vdab148_suppl_Supplementary_Figure_S11 [file vdab148_suppl_supplementary_figure_s11.jpeg]

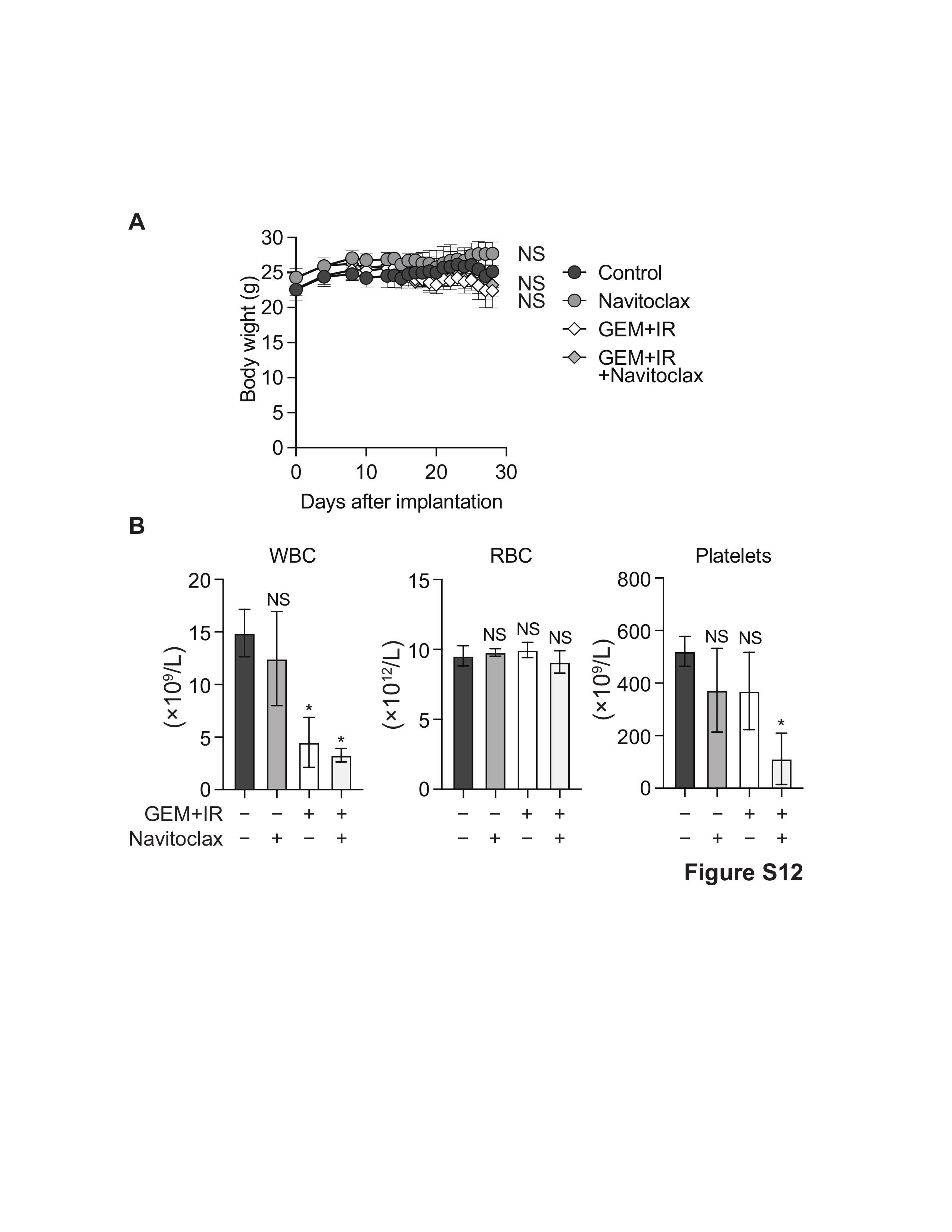

Supplement: vdab148_suppl_Supplementary_Figure_S12 [file vdab148_suppl_supplementary_figure_s12.jpeg]

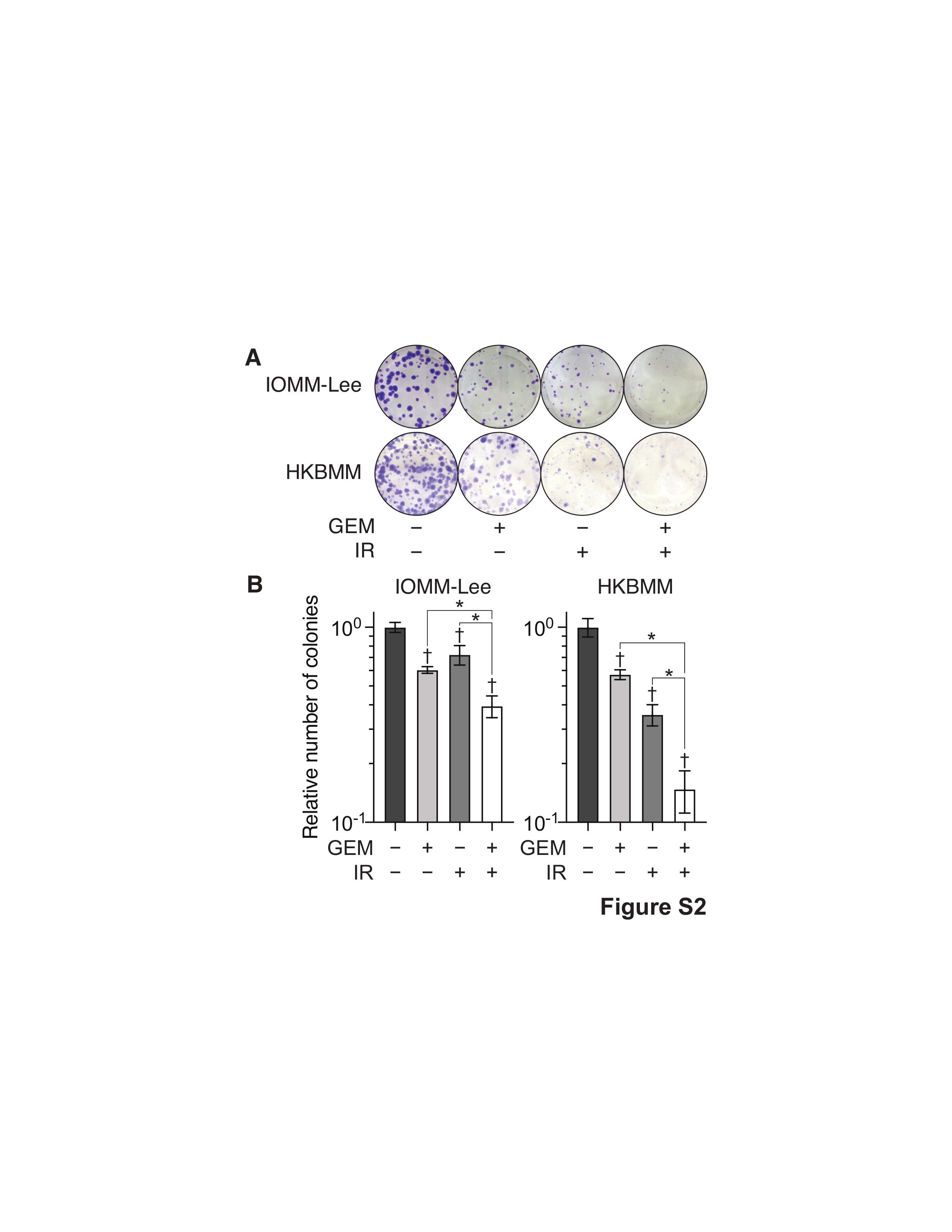

Supplement: vdab148_suppl_Supplementary_Figure_S2 [file vdab148_suppl_supplementary_figure_s2.jpeg]

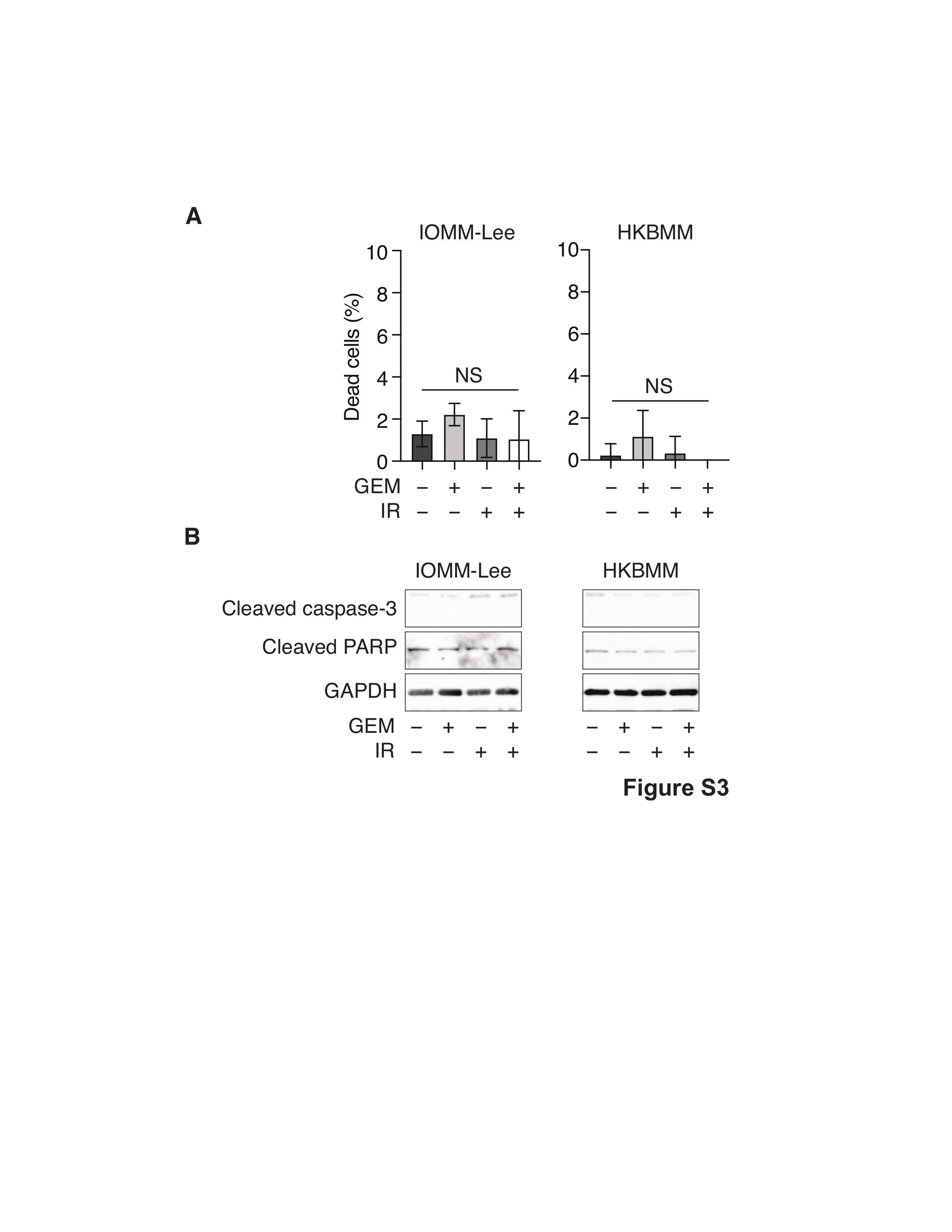

Supplement: vdab148_suppl_Supplementary_Figure_S3 [file vdab148_suppl_supplementary_figure_s3.jpeg]

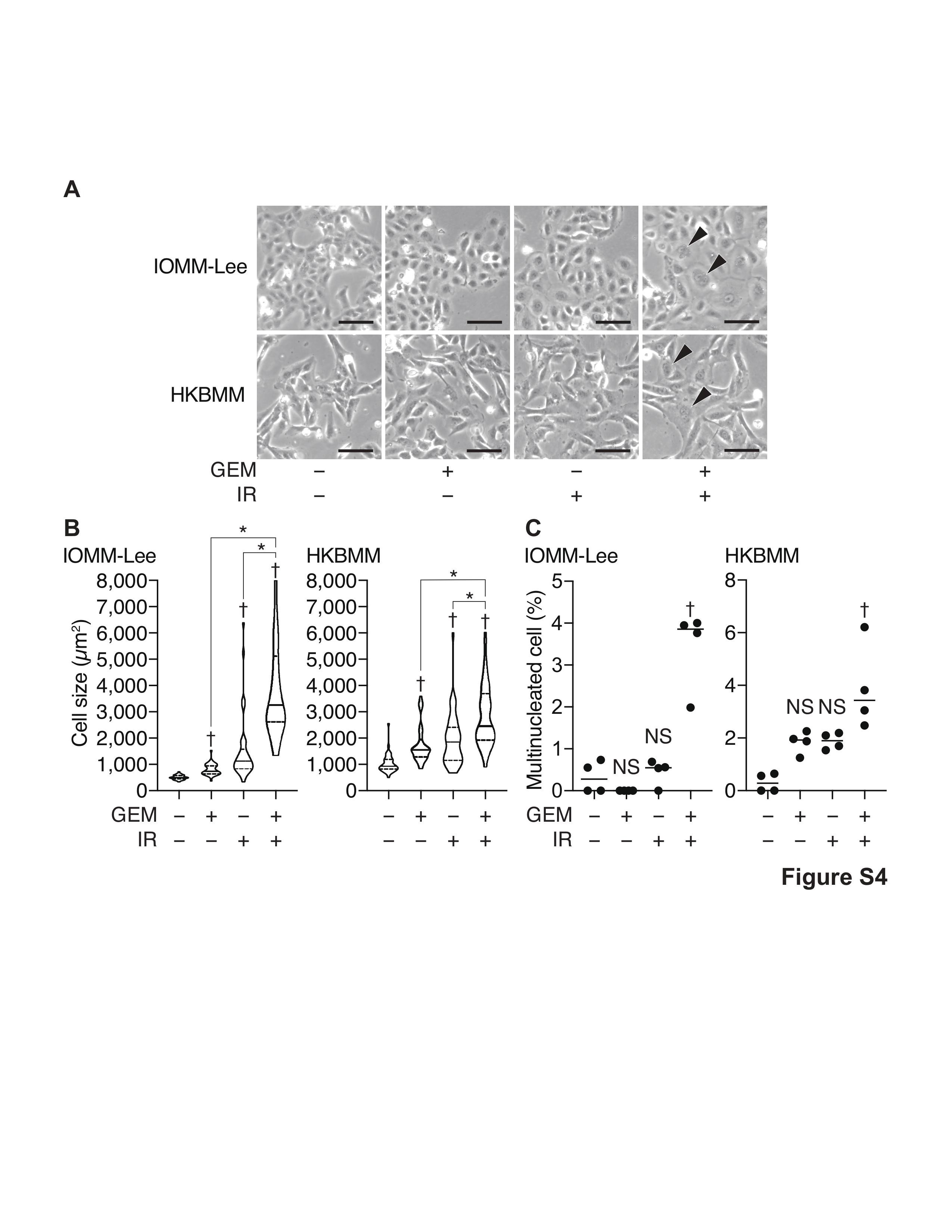

Supplement: vdab148_suppl_Supplementary_Figure_S4 [file vdab148_suppl_supplementary_figure_s4.jpeg]

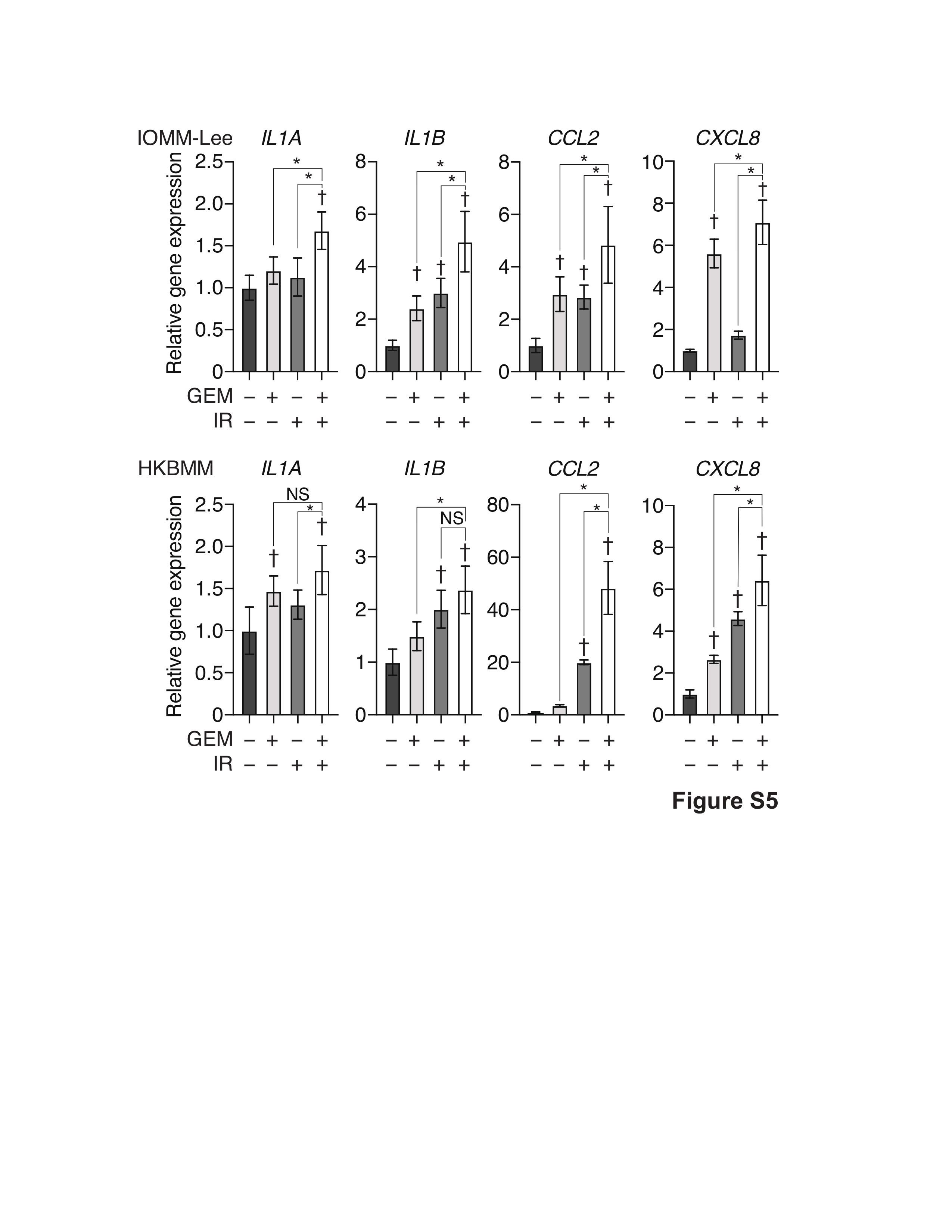

Supplement: vdab148_suppl_Supplementary_Figure_S5 [file vdab148_suppl_supplementary_figure_s5.jpeg]

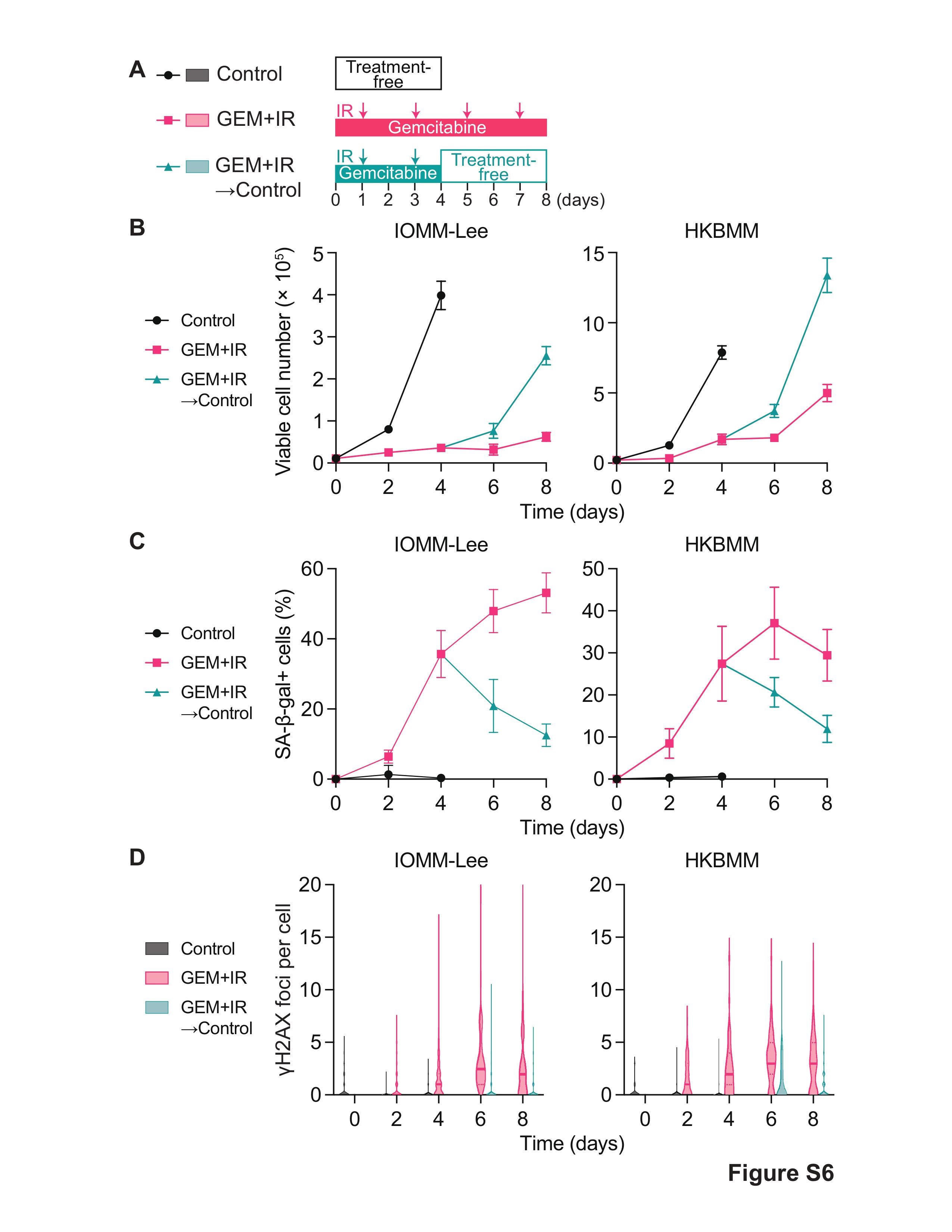

Supplement: vdab148_suppl_Supplementary_Figure_S6 [file vdab148_suppl_supplementary_figure_s6.jpeg]

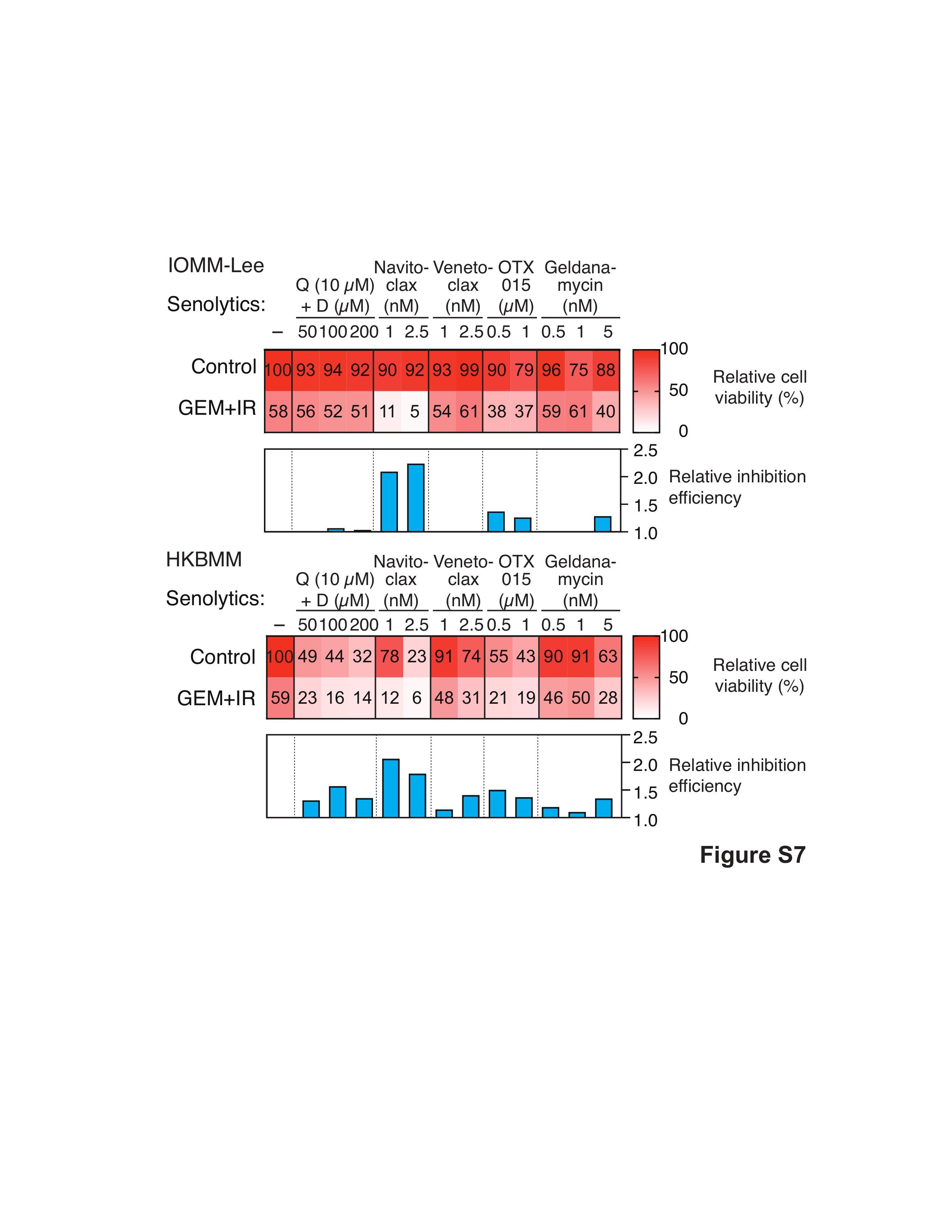

Supplement: vdab148_suppl_Supplementary_Figure_S7 [file vdab148_suppl_supplementary_figure_s7.jpeg]

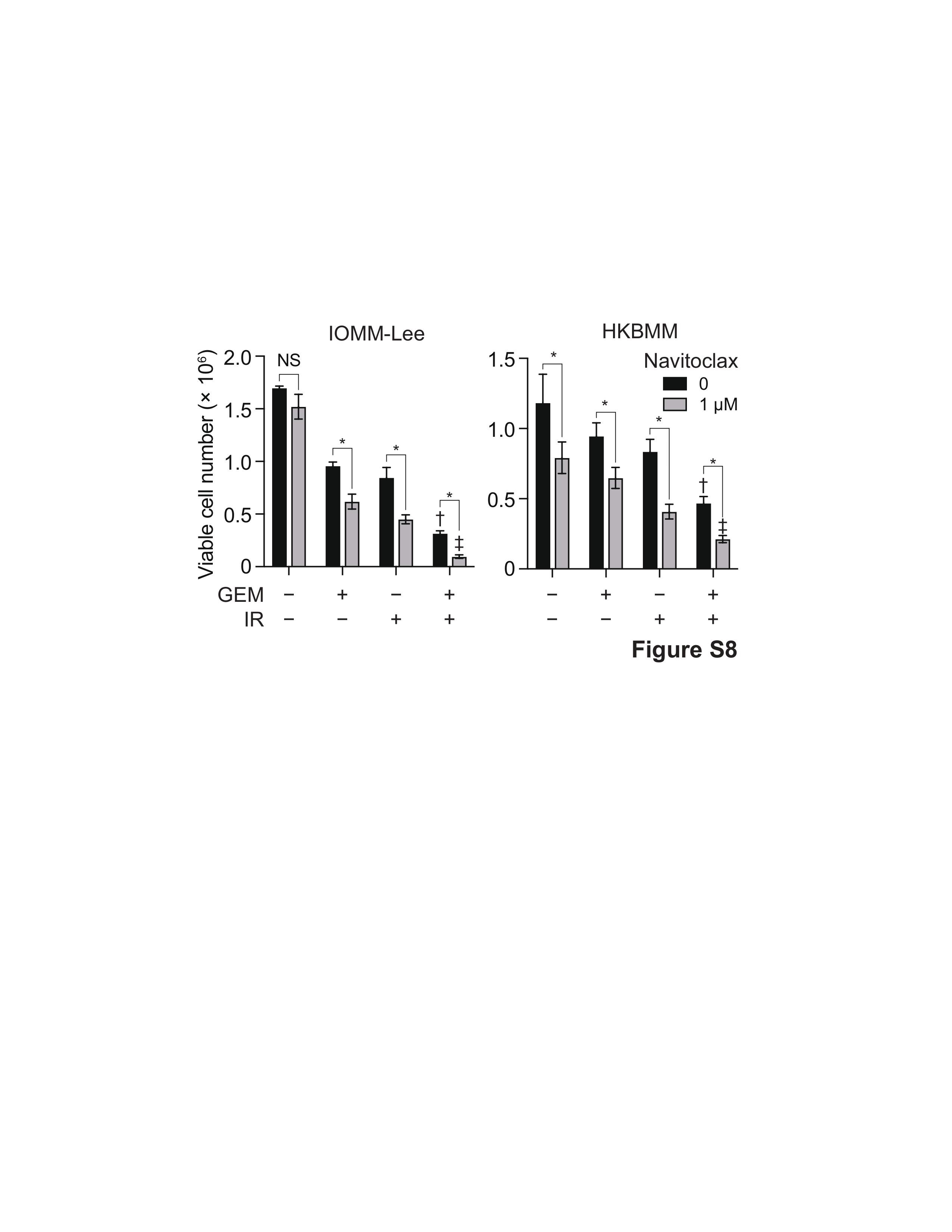

Supplement: vdab148_suppl_Supplementary_Figure_S8 [file vdab148_suppl_supplementary_figure_s8.jpeg]

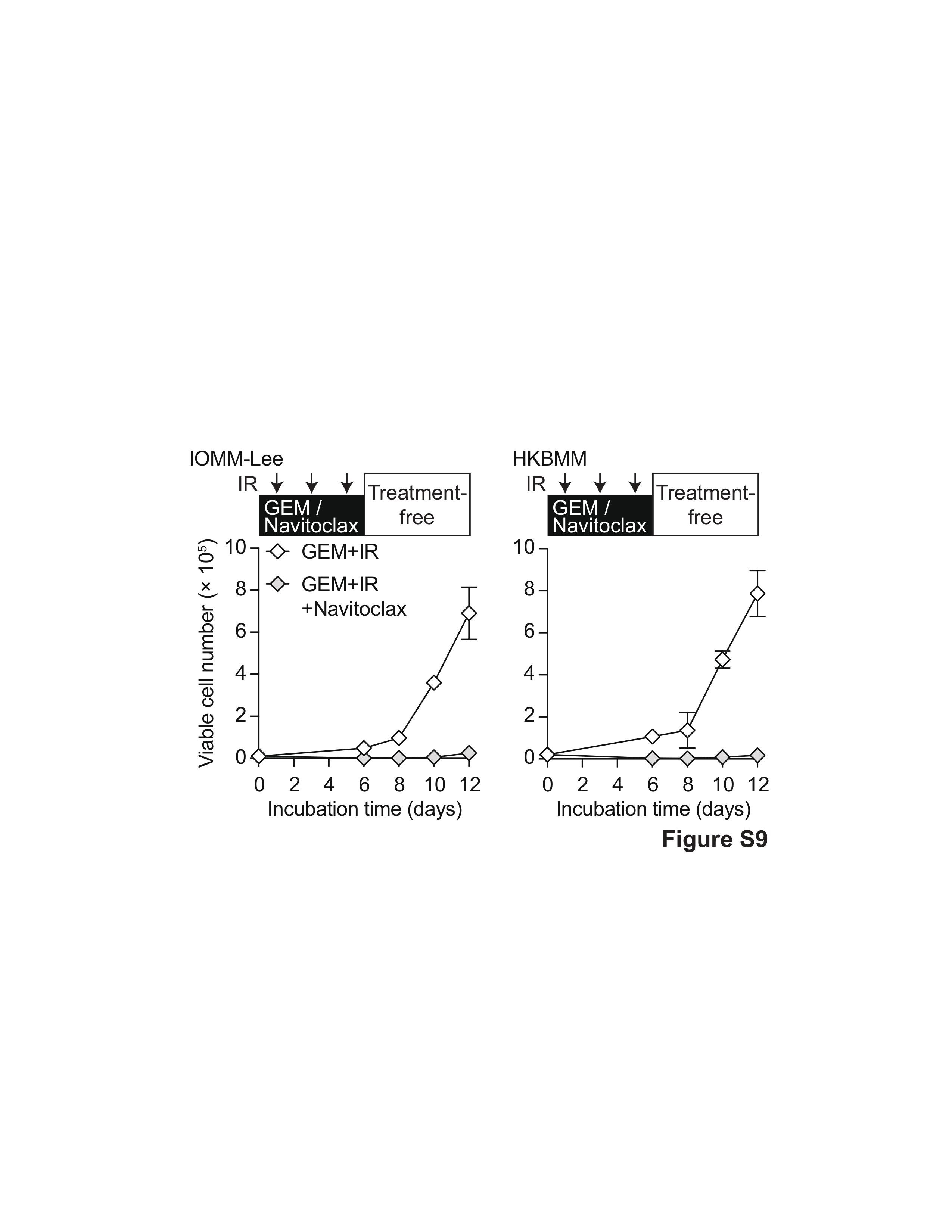

Supplement: vdab148_suppl_Supplementary_Figure_S9 [file vdab148_suppl_supplementary_figure_s9.jpeg]
