# Supplementary material for: Gemcitabine radiosensitization primes irradiated malignant meningioma cells for senolytic elimination by navitoclax
Source: Neurooncol Adv. 2021 Oct 8;3(1):vdab148. doi: 10.1093/noajnl/vdab148 (PMC8577526; doi:10.1093/noajnl/vdab148)
Supplement: vdab148_suppl_Supplementary_Material [file vdab148_suppl_supplementary_material.docx]

**Supplementary Methods**

*Antibodies and reagents*

Anti-cleaved caspase-3 (#9661), anti-glyceraldehyde 3-phosphate dehydrogenase (#5174, GAPDH), anti-phospho-histone H2A.X (#9718, ser139, γH2AX), anti-Ki-67 (#9027), and anti-cleaved PARP (#9541) antibodies were purchased from Cell Signaling Technology (Beverly, MA, USA). An anti-Bcl-xL antibody (10783-1-AP) was purchased from ProteinTech (Rosemont, IL, USA). An anti-Bcl-2 antibody (sc-7382) was purchased from Santa Cruz Biotechnologies (Dallas, TX, USA). Gemcitabine was purchased from Fujifilm Wako Pure Chemical Corporation (Osaka, Japan) and dissolved in distilled water to prepare 1 mM and 8 mg/mL stock solutions for *in vitro* and *in vivo* studies, respectively. Navitoclax (ABT-263) was purchased from Chemscene (Monmouth Junction, NJ, USA) and dissolved in DMSO to prepare 100 mM and 100 mg/mL stock solutions for *in vitro* and *in vivo* studies, respectively. N-acetyl-cysteine (NAC) was purchased from Sigma-Aldrich (St. Louis, MO, USA) and dissolved into DMSO to prepare a 5 M stock solution. Quercetin, dasatinib, OTX015, and A-1331852 were purchased from Cayman Chemicals (Ann Arbor, MI, USA) and dissolved in DMSO to prepare 10, 10, 1, and 1 mM stock solutions, respectively. Venetoclax (ABT-199) was purchased from LC Laboratories (Woburn, MA, USA) and dissolved into DMSO to prepare a 50 mM stock solution. Geldanamycin was purchased from Toronto Research Chemicals (Toronto, Canada) and dissolved into DMSO to prepare a 10 mM stock solution.

*Cell viability assay*

Cell viability was assessed using the trypan blue dye exclusion assay (Figures 1, 3, 5, and S3) or WST-8 assay (Figures S1, S6, and S7) using Cell Counting Kit-8 (Dojindo Laboratories, Kumamoto, Japan) as previously described [^1^](#_ENREF_1)^,^[^2^](#_ENREF_2). Briefly, after being trypsinized and suspended into phosphate-buffered saline (PBS), cells were stained with 0.2% trypan blue, and viable and dead cells were identified by their ability and inability, respectively, to exclude trypan blue. The percentage of dead cells was defined as 100 × the number of dead cells / (the number of viable cells + the number of dead cells). The WST-8 reagent was added to the culture medium of cells grown on 96-well cell culture plates, and cells were incubated at 37ºC for 1–3 hours. Absorbance at 450 nm was measured using a microplate reader (Model 680, Bio-Rad, Hercules, CA, USA). Relative cell viability was calculated as a percentage of the absorbance of treated samples relative to that of control samples.

*Western blotting*

Western blotting was conducted as previously described [^2-4^](#_ENREF_2). Cells were washed with ice-cold PBS and lysed in RIPA buffer [10 mM Tris/HCl (pH 7.4), 0.1% sodium dodecyl sulfate (SDS), 0.1% sodium deoxycholate, 1% NP-40, 150 mM NaCl, 1 mM EDTA, 1.5 mM Na_3_VO_4_, 10 mM NaF, 10 mM sodium pyrophosphate, 10 mM sodium β-glycerophosphate, and 1% protease inhibitor cocktail set III (Wako Pure Chemical Industries, Ltd., Osaka, Japan)]. This was followed by the immediate addition of the same volume of Laemmli buffer 2× [125 mM Tris/HCl (pH 6.8), 4% SDS, 10% glycerol] and boiling at 95°C for 10 min. Protein concentrations were measured using a BCA protein assay kit (Thermo Fisher Scientific, Waltham, MA, USA). Samples containing equal amounts of protein were resolved by SDS-polyacrylamide gel electrophoresis and transferred to polyvinylidene fluoride membranes. Membranes were probed with a primary antibody followed by a horseradish peroxidase (HRP)-conjugated secondary antibody as recommended by the manufacturer of each antibody. Specific bands were visualized using Immobilon Western Chemiluminescent HRP Substrate (Merck Millipore, Billerica, MA, USA) and detected semi-quantitatively by the ChemiDoc Touch Imaging System (Bio-Rad).

*Immunocytochemistry for γH2AX*

Cells grown on glass coverslips were fixed with 4% paraformaldehyde for 5 minutes, permeabilized with 0.2% Triton X-100 for 5 minutes, and then blocked with 1% BSA for 10 minutes. Anti-phospho-histone H2A.X (#9718, ser139, γH2AX, Cell Signaling Technologies) was added and incubated at room temperature for 1 hour. After washing with PBS, AlexaFluor-568-conjugated anti-rabbit immunoglobulin (A-11011, Thermo Fischer Scientific) was added and incubated at room temperature for 30 minutes. Nuclei were counterstained with Hoechst 33342 (20 µg/mL, H3570, Thermo Fischer Scientific). Fluorescent images were obtained using a BZ-X700 microscope (Keyence, Osaka, Japan). The number of γH2AX foci was quantified in at least 60 cells per condition, and the frequency distribution of the number of γH2AX foci was shown as a violin plot.

*Immunohistochemistry*

Excised tissues were fixed with 4% paraformaldehyde at 4ºC overnight, embedded in paraffin, and then cut into 4-µm-thick sections. After deparaffinization and rehydration, sections were treated with 3% hydrogen peroxide for 10 min. Antigens were retrieved by a heat treatment in 0.1 M Tris-HCl buffer (pH 9.0). Slides were incubated with the primary antibody at 4ºC overnight. Immunostaining was performed using Histofine Simple Stain MAX-PO (Nichirei Biosciences, Tokyo, Japan) and ImmPACT DAB (Vector Laboratories, Burlingame, CA).

*Senescence-associated beta-galactosidase (SA-β-gal) staining*

The SA-β-gal stain was performed using a Cellular Senescence Assay Kit (Cell Biolabs, San Diego, CA, USA) according to the manufacturer’s instructions. Briefly, for cultured cells, cells were fixed with 0.25% glutaraldehyde for 5 minutes and stained with the Cell Staining Working Solution at 37ºC for 1 day. Bright-field images were obtained using a BZ-X700 microscope (Keyence). More than 50 cells were counted to calculate the percentage of SA-β-gal-positive cells. Regarding subcutaneous tumors, excised tumors were fixed with 4% paraformaldehyde for 10–20 minutes, washed with PBS, and then incubated in Cell Staining Working Solution at 37ºC for 1 day. After dehydrating with 30% sucrose solution for 2 days, tissues were embedded in Tissue-Tek OCT compound (Sakura Finetek Japan, Tokyo, Japan) and cut into 10-µm-thick sections. Three representative images of each tumor were obtained using a light microscope (BX63, Olympus, Tokyo, Japan), and the percentage of the SA-β-gal-positive area was quantified using ImageJ (version 2.1.0) software [^5^](#_ENREF_5).

*Measurement of intracellular ROS*

Cells were incubated in culture medium containing 2.5 μM CellRox Green (Thermo Fischer Scientific) at 37°C for 60 min, and washed twice with PBS. After trypsinization, cells were fixed with 4% paraformaldehyde and washed with PBS. Cells suspended in PBS were then subjected to a flow cytometric analysis on FACSCanto II Flow Cytometer (BD Biosciences, San Jose, CA, USA), and data were analyzed using FlowJo software version 10 (BD Biosciences). Unstained cells were used to select the threshold for CellRox-positive cells.

*Measuring cell size*

To measure the size of cultured cells, phase-contrast images were obtained using a BZ-X700 microscope (Keyence), and the area of individual cells was measured using ImageJ (version 2.1.0) software.

*Colony formation assay*

A colony formation assay was performed as described previously [^4^](#_ENREF_4)^,^[^6^](#_ENREF_6). In brief, cells were seeded at a low colony-forming density (500 cells/6-well plate for IOMM-Lee and 1,000 cells/6-well plate for HKBMM) and cultured with or without 3 and 2 nM gemcitabine for IOMM-Lee and HKBMM, respectively, for 6 days followed by a culture in the absence of gemcitabine for 4–6 days. During the gemcitabine treatment, cells were irradiated with 1 and 2 Gy for IOMM-Lee and HKBMM, respectively, on the 1st, 3rd, and 5th days of treatment. Cells were then fixed with paraformaldehyde (4% w/v), followed by staining with crystal violet (0.1% w/v).

*Gene silencing by siRNA*

siRNAs against human Bcl-xL (*BCL2L1*: #1 HSS141361, #2 HSS141363) and Medium GC Duplex #2 of Stealth RNAi siRNA Negative Control Duplexes (non-targeting control, siControl) were purchased from Thermo Fisher Scientific. IOMM-Lee and HKBMM were transfected with siRNA against Bcl-xL or with control RNA (siControl) using Lipofectamine RNAiMAX (Thermo Fisher Scientific) according to the manufacturer’s instructions.

*Quantitative reverse transcription PCR*

RNA was extracted from cells using TRIzol reagent (Thermo Fisher Scientific) and then reverse-transcribed into cDNA using the PrimeScript II 1st strand cDNA Synthesis Kit (Takara Bio, Kusatsu, Japan). Quantitative PCR was performed with a Thunderbird SYBR qPCR Mix (Toyobo, Osaka, Japan) using CFX96 C1000 Thermal Cycler (Bio-Rad). mRNA levels were calculated using the comparative *C*_T_ method [^7^](#_ENREF_7) and normalized to the values of the *ACTB* (β-actin) gene. The sequences of gene-specific primer sets are listed in Table S1.

*Complete blood count*

Blood was collected via the tail vein of mice, and the number of blood cells was measured using the VetScan HM5 Hematology Analyzer (Zoetis, Parsippany, NJ, USA).

**References**

**1.** Suzuki S, Yamamoto M, Sanomachi T, et al. Doxazosin, a Classic Alpha 1-Adrenoceptor Antagonist, Overcomes Osimertinib Resistance in Cancer Cells via the Upregulation of Autophagy as Drug Repurposing. *Biomedicines.* 2020; 8(8):273.

**2.** Yamamoto M, Suzuki S, Togashi K, et al. AS602801 Sensitizes Ovarian Cancer Stem Cells to Paclitaxel by Down-regulating MDR1. *Anticancer Res.* 2019; 39(2):609-617.

**3.** Togashi K, Okada M, Yamamoto M, et al. A Small-molecule Kinase Inhibitor, CEP-1347, Inhibits Survivin Expression and Sensitizes Ovarian Cancer Stem Cells to Paclitaxel. *Anticancer Res.* 2018; 38(8):4535-4542.

**4.** Yamamoto M, Suzuki S, Togashi K, et al. AS602801, an Anticancer Stem Cell Candidate Drug, Reduces Survivin Expression and Sensitizes A2780 Ovarian Cancer Stem Cells to Carboplatin and Paclitaxel. *Anticancer Res.* 2018; 38(12):6699-6706.

**5.** Schneider CA, Rasband WS, Eliceiri KW. NIH Image to ImageJ: 25 years of image analysis. *Nat Methods.* 2012; 9(7):671-675.

**6.** Seino M, Okada M, Sakaki H, et al. Time-staggered inhibition of JNK effectively sensitizes chemoresistant ovarian cancer cells to cisplatin and paclitaxel. *Oncol Rep.* 2016; 35(1):593-601.

**7.** Schmittgen TD, Livak KJ. Analyzing real-time PCR data by the comparative C(T) method. *Nat Protoc.* 2008; 3(6):1101-1108.

**Supplementary Table S1**

Sequences of primers for qPCR.

Gene name Forward Reverse

*IL1A* GCTGAAGGAGATGCCTGAGATA ACAAGTTTGGATGGGCAACTG

*IL1B* AACAGGCTGCTCTGGGATTC AGTCATCCTCATTGCCACTGT

*CCL2* CCCAGTCACCTGCTGTTATAAC AGATCTCCTTGGCCACAATG

*CXCL8* AAGAAACCACCGGAAGGAAC ACTCCTTGGCAAAACTGCAC

*ACTB* CCCATGCCATCCTGCGTCTG CGTCATACTCCTGCTTGCTG

**Supplementary Figure Legends**

**Figure S1 Combined effects of gemcitabine and ionizing radiation on meningioma cells.**

IOMM-Lee (A) and HKBMM (B) cells plated on 96-well plates in duplicate (500 IOMM-Lee cells and 1,000 HKBMM cells per each well) were incubated with gemcitabine (0, 2, 3, 4, or 5 nM) for 3 days and irradiated by X-ray (0, 1, 2, 4, 6, or 8 Gy) on day 2. Treated cells were incubated for another 3 days without gemcitabine, and then subjected to a WST assay. The percentages of relative average absorbance to the Control (GEM 0 and IR 0) are indicated in squares and shown as heat maps.

**Figure S2 Effects of gemcitabine and ionizing radiation on colony-forming activity in malignant meningioma cells.**

Cells plated at colony-forming density on 6-well plates in 6 replicates (500 cells per well for IOMM-Lee and 1,000 cells per well for HKBMM) were incubated without or with gemcitabine (3 nM for IOMM-Lee and 2 nM for HKBMM) for 6 days and not irradiated or irradiated three times by X-ray (1 Gy for IOMM-Lee and 2 Gy for HKBMM) on days 1, 3, and 5, followed by an incubation without any treatments for another 4–6 days. Colonies were stained with crystal violet (A), the number of colonies was counted, and the relative number of colonies was calculated by dividing the number of colonies in each well by the average number of colonies in the Control (GEM− and IR−). (B). Values are shown as means ± SD. *P*-values were calculated by a one-way ANOVA with Tukey’s *post-hoc* test. *, *P* < 0.05. †, *P* < 0.05 versus the Control (GEM− and IR−).

**Figure S3 Effects of gemcitabine and ionizing radiation on cell death in malignant meningioma cells.**

IOMM-Lee and HKBMM cells plated on 6-well plates (IOMM-Lee: 2 × 10^5^; HKBMM: 4 × 10^5^ cells per well for a cell death assay) or 6-cm dishes (for an immunoblot analysis) were incubated without or with gemcitabine (3 nM for IOMM-Lee and 2 nM for HKBMM) for 6 days and not irradiated or irradiated 3 times by X-ray (1 Gy for IOMM-Lee and 2 Gy for HKBMM) on days 1, 3, and 5. (A) Percentages of dead cells on day 6. Dead cells were counted in 6 replicates. Values are shown as means ± SD. *P*-values were calculated by the Brown-Forsythe and Welch ANOVA tests. NS, *P* ≥ 0.05. (B) Western blot analysis of the indicated proteins on day 6. GEM, gemcitabine. IR, ionizing radiation.

**Figure S4 Enlargement and multinucleation of malignant meningioma cells by gemcitabine and ionizing radiation.**

IOMM-Lee and HKBMM cells were incubated without or with gemcitabine (3 nM for IOMM-Lee and 2 nM for HKBMM) for 6 days and not irradiated or irradiated 3 times by X-ray (1 Gy for IOMM-Lee and 2 Gy for HKBMM) on days 1, 3, and 5. (A) Representative phase-contrast images on day 6. Arrowheads, multinucleated cells. Scale bars, 100 µm. (B) Quantification of cell size. The size of 40 cells per group was measured and is shown as violin plots (line, median; dotted lines, quartile). (C) Percentage of multinucleated cells. More than 100 cells per group were counted in 4 replicates. Bar, median. *P*-values were calculated by the Kruskal-Wallis test with Dunn’s multiple comparisons test. *, *P* < 0.05. †, *P* < 0.05 and NS, *P* ≥ 0.05 versus the Control (GEM− and IR−). GEM, gemcitabine. IR, ionizing radiation.

**Figure S5 Effects of gemcitabine and ionizing radiation on the expression of genes related to the senescence-associated secretory phenotype in malignant meningioma cells.**

IOMM-Lee and HKBMM cells were incubated without or with gemcitabine (3 nM for IOMM-Lee and 2 nM for HKBMM) for 4 days and not irradiated or irradiated twice by X-ray (1 Gy for IOMM-Lee and 2 Gy for HKBMM) on days 1 and 3, followed by a RT-qPCR analysis. GEM, gemcitabine. IR, ionizing radiation. The expression of each gene was normalized to the expression of the *ACTB* gene. Values are shown as means ± SD (n = 6, each group). *P*-values were calculated by a one-way ANOVA with Sidak’s *post-hoc* test. *, *P* < 0.05. †, *P* < 0.05 and NS, *P* ≥ 0.05 versus the Control (GEM− and IR−).

**Figure S6 Recovery of malignant meningioma cells from cell growth suppression and senescence after gemcitabine and ionizing radiation.**

IOMM-Lee and HKBMM cells plated on 6-well plates (1 × 10^4^ cells per well for IOMM-Lee and 2 × 10^4^ cells per well for HKBMM) were incubated without any treatments for 4 days (Control group, shown in black) or with gemcitabine for 8 days (3 nM for IOMM-Lee and 2 nM for HKBMM) in combination with ionizing radiation (1 Gy for IOMM-Lee and 2 Gy for HKBMM) on days 1, 3, 5, and 7 (GEM+IR group, shown in magenta). Alternatively, these cells were incubated with gemcitabine for 4 days in combination with ionizing radiation on days 1 and 3 and then incubated for a further 4 days without any treatments (GEM+IR→Control group, shown in green). Schema of the protocol (A). The viable cell number (B, in triplicate), the percentage of SA-β-gal (C, in 4 replicates), and the number of γH2AX foci (> 60 cells were counted) were evaluated every 2 days. In (B) and (C), values are shown as means ± SD. In (D), data are shown as violin plots (line, median; dotted lines, quartile).

**Figure S7 Enhancement of effects of gemcitabine and ionizing radiation by senolytics.**

IOMM-Lee and HKBMM cells plated on 96-well plates in duplicate (2,000 cells per each well) were untreated (Control) or treated with gemcitabine for 4 days (3 nM for IOMM-Lee and 2 nM for HKBMM) in combination with ionizing radiation twice (1 Gy for IOMM-Lee and 2 Gy for HKBMM) on days 1 and 3 (GEM+IR) in the absence (−) or presence of the indicated senolytics, and cell viability was examined using a WST-8 assay. The percentages of average relative cell viability to the Control (GEM−, IR−, and senolytics−) are indicated in each square and shown as heat maps (upper). The relative inhibition efficiency values of each senolytic are shown as bar graphs (lower). Inhibition efficiency for each senolytic and control (senolytics−) was calculated by the following formula: 1 − [GEM+IR]/[Control], where [GEM+IR] and [Control] are the relative cell viability values of GEM and IR-treated and untreated (Control) cells, respectively. The relative inhibition efficiency of each senolytic was calculated by dividing the inhibition efficiency of each senolytic by that of untreated cells (senolytics−). Q, quercetin; D, dasatinib.

**Figure S8 Combined effects of navitoclax with gemcitabine and ionizing radiation on meningioma cell growth.**

IOMM-Lee and HKBMM cells plated on 6-well plates in triplicate (IOMM-Lee: 2 ×10^4^; HKBMM: 4 × 10^4^ cells per well) were incubated without or with gemcitabine (3 nM for IOMM-Lee and 2 nM for HKBMM) for 4 days and not irradiated or irradiated twice by X-ray (1 Gy for IOMM-Lee and 2 Gy for HKBMM) on days 1 and 3 in the absence or presence of navitoclax (1 µM), and then subjected to a cell viability assay. GEM, gemcitabine. IR, ionizing radiation. Values are shown as means ± SD. *P*-values were calculated by a one-way ANOVA with Tukey’s *post-hoc* test. NS, *P* ≥ 0.05. *, *P* < 0.05. †, *P* < 0.05, significantly different from all other groups without the navitoclax treatment (Control/Nav0, GEM+Nav0, and IR+Nav0). ‡, *P* < 0.05, significantly different from all other groups with the navitoclax treatment (Control/Nav1, GEM+Nav1, and IR+Nav1).

**Figure S9 Effects of navitoclax on the regrowth of meningioma cells after gemcitabine and ionizing radiation.** IOMM-Lee and HKBMM cells plated on 6-well plates in triplicate (IOMM-Lee: 1 ×10^4^; HKBMM: 2 × 10^4^ cells per well) were incubated with gemcitabine (3 nM for IOMM-Lee and 2 nM for HKBMM) for 6 days in combination with ionizing radiation (1 Gy for IOMM-Lee and 2 Gy for HKBMM) on days 1, 3, and 5 in the absence or presence of navitoclax (1 µM), and then incubated without any treatments for a further 6 days. Viable cell numbers were serially assessed. Values are shown as means ± SD.

**Figure S10 Roles of Bcl-xL in the survival of malignant meningioma cells treated with gemcitabine and ionizing radiation.**

IOMM-Lee and HKBMM cells were incubated without or with gemcitabine (3 nM for IOMM-Lee and 2 nM for HKBMM) for 4 days and not irradiated or irradiated twice by X-ray (1 Gy for IOMM-Lee and 2 Gy for HKBMM) on days 1 and 3, and then subjected to a Western blot analysis (A). IOMM-Lee and HKBMM cells were transfected with siControl or siBcl-xL (#1 or #2). Transfected cells were harvested 4 days after transfection and subjected to Western blotting (B). Alternatively, cells grown in 96-well plates in 5 replicates were transfected with siControl or siBcl-xL (#1 and #2). On the following day, transfected cells were untreated (Control) or treated with gemcitabine (3 nM for IOMM-Lee and 2 nM for HKBMM) in combination with X-ray irradiation twice on days 1 day 3 (GEM + IR), followed by a WST-8 assay (C). GEM, gemcitabine. IR, ionizing radiation. Values were shown as means ± SD. *P*-values were calculated by a one-way ANOVA with Tukey’s *post-hoc* test. *, *P* < 0.05. †, *P* < 0.05 versus the untreated siControl. ‡, *P* < 0.05 versus the GEM+IR-treated siControl.

**Figure S11 Effects of A-1331852, a selective inhibitor of Bcl-xL, combined with gemcitabine and ionizing radiation on malignant meningioma cells.**

IOMM-Lee and HKBMM cells plated on 6-well plates in 3 replicates (2 × 10^4^ cells per well for IOMM-Lee and 4 × 10^4^ cells per well for HKBMM) were incubated for 4 days without or with gemcitabine (3 nM for IOMM-Lee and 2 nM for HKBMM) in combination with ionizing radiation (1 Gy for IOMM-Lee and 2 Gy for HKBMM) on days 1 and 3 in the absence or presence of A-1331852 (1 µM), and were then subjected to a cell viability assay to assess the viable cell number (A) and percentage of dead cells (B). Values are shown as means ± SD. *P*-values were calculated by a one-way ANOVA with Tukey’s *post-hoc* test. *, *P* < 0.05. †, *P* < 0.05 versus the Control (GEM+IR− and A-1331852−).

**Figure S12 Adverse effects of navitoclax combined with gemcitabine and ionizing radiation.** This experiment is the same as that shown in Figure 6. Mice were treated with navitoclax (100 mg/kg, oral gavage, every day) or gemcitabine (10 mg/kg, intraperitoneal injection) in combination with ionizing radiation (1 Gy) (3 times a week, arrows) (GEM+IR), both (GEM+IR+Navitoclax), or vehicle (Control). The body weights of mice were measured (A) and white blood cell (WBC), red blood cell (RBC), and platelet counts in blood were counted 28 days after implantation. Values are shown as means ± SD. n = 4, for each group. *P*-values were calculated by a one-way ANOVA with Dunnett’s multiple comparisons test. *, *P* < 0.05 and NS, *P* ≥ 0.05 versus the Control (no treatment).
